# Borate Hydrides as a New Material Class: Structure, Computational Studies, and Spectroscopic Investigations on Sr_5_(BO_3_)_3_H and Sr_5_(^11^BO_3_)_3_D

**DOI:** 10.1002/chem.202002273

**Published:** 2020-08-17

**Authors:** Thomas Wylezich, Renaud Valois, Markus Suta, Alexander Mutschke, Clemens Ritter, Andries Meijerink, Antti J. Karttunen, Nathalie Kunkel

**Affiliations:** ^1^ Institut für Anorganische Chemie Georg-August-Universität Göttingen Tammannstr. 4 37077 Göttingen Germany; ^2^ Woehler Research Institute for Sustainable Chemistry (WISCh) Georg-August-University Goettingen Tammannstr. 2 37077 Goettingen Germany; ^3^ Chair of Inorganic Chemistry with Focus on Novel Materials Department of Chemistry Technical University Munich Lichtenbergstr. 4 85748 Garching Germany; ^4^ UCCS-UMR CNRS 8181 Université d'Artois, Faculté de, Sciences Jean Perrin Rue Jean Souvraz 62300 Lens France; ^5^ Condensed Matter and Interfaces Debye Institute for, Nanomaterials Science Department of Chemistry Utrecht University Princetonplein 1 3584 CC Utrecht Netherlands; ^6^ Institut Laue-Langevin 71 Avenue des Martyrs 38042 Grenoble France; ^7^ Department of Chemistry and Materials Science Aalto University P.O. Box 16100 FI-00076 Aalto Finland

**Keywords:** borate, europium, hydride, luminescence, neutron diffraction

## Abstract

The unprecedented borate hydride Sr_5_(BO_3_)_3_H and deuteride Sr_5_(^11^BO_3_)_3_D crystallizing in an apatite‐related structure are reported. Despite the presence of hydride anions, the compound decomposes only slowly in air. Doped with Eu^2+^, it shows broad‐band orange‐red emission under violet excitation owing to the 4f^6^5d–4f^7^ transition of Eu^2+^. The observed ^1^H NMR chemical shift is in good agreement with previously reported ^1^H chemical shifts of ionic metal hydrides as well as with quantum chemical calculations and very different from ^1^H chemical shifts usually found for hydroxide ions in similar materials. FTIR and Raman spectroscopy of different samples containing ^1^H, ^2^H, ^nat^B, and ^11^B combined with calculations unambiguously prove the absence of hydroxide ions and the sole incorporation of hydride ions into the borate. The orange‐red emission obtained by doping with Eu^2+^ shows that the new compound class might be a promising host material for optical applications.

## Graphical abstract

**Enlighten me**! The unprecedented borate hydride (deuteride) Sr_5_(BO_3_)_3_H (Sr_5_(BO_3_)_3_D) is synthesized mechanochemically and its structure is determined by neutron diffraction. DFT calculations, NMR, IR, and Raman measurements unambiguously confirm the successful synthesis. An orange‐red phosphor is obtained upon doping with Eu^2+^.

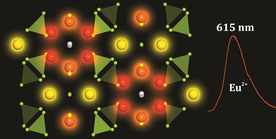

## Introduction

The chemical and physical properties of ionic materials usually do not only depend on the cation chemistry, but also to a large extent on the anion chemistry and combinations of different anions.^1^, ^2^, ^3^ Tuning the anion chemistry allows the material properties to be optimized by taking advantage of the different anion radii, electronegativities, polarizabilities, and charges. Recently, especially in the field of mixed anionic hydride‐containing compounds, there was a number of successful proofs of this chemical concept leading to related interesting properties.^1^, ^3^, ^4^ For instance, fast hydride conduction or electronic conductivity was found in oxide hydrides.^5^, ^6^, ^7^, ^8^, ^9^, ^10^ The lability of hydride ligands in oxide hydrides allowed the synthesis of other compounds more easily, such as oxide nitrides, which are, for example, of great interest as catalysts, but may be difficult to access by other preparation methods.^11^, ^12^ Hydride anions were also found in phosphates crystallizing in the apatite structure.^13^, ^14^ Furthermore, oxide hydrides,^15^ halide hydrides,^16^, ^17^, ^18^, ^19^ as well as even silicate hydrides^20^, ^21^ have been recently used as host compounds for phosphors activated with lanthanides. The strong polarizability of the hydride anion, which generally leads to low energetic 5d–4f emission bands of Eu^2+^ combined with the variation of the other anions, raises the potential for chemical tuning of their desired properties. In addition to divalent europium, it was also shown that hydride has an influence on the excitation spectra of Tb^3+^.^15^

Besides compound classes such as nitrides and oxide nitrides,^22^, ^23^, ^24^ silicates,^25^ or oxides^26^, ^27^ also borates and borate halides are of great interest as optical materials, either for second harmonic generation applications as the respective solids often crystallize in non‐centrosymmetric space groups or, additionally, as host materials for embedded luminescent ions.^28^, ^29^, ^30^, ^31^, ^32^, ^33^, ^34^, ^35^, ^36^, ^37^, ^38^, ^39^ Examples include lanthanide‐doped Sr_5_(BO_3_)_3_F:Ln (Ln=Eu, Ce)^32^, ^33^ as well as Sr_5_(BO_3_)_3_Cl:Eu^2+[34]^ and the fully substituted variant Eu_5_(BO_3_)_3_F^35^ is known. Violet or blue‐light excited red‐emitting phosphors remain a challenge for the development of white light‐emitting diodes (wLEDs), but continuous efforts are being made in this direction.^40^, ^41^ Encouraged by the recent discovery of fully substituted phosphate and silicate hydrides^14^, ^20^ and taking the known borate fluoride Sr_5_(BO_3_)_3_F^42^ as a starting model, we aimed at the preparation of a hitherto unknown type of compound, the borate hydride Sr_5_(BO_3_)_3_H. Although attempts to obtain the compound by classical solid‐state techniques were so far unsuccessful, the desired compound could be prepared by a mechanochemical approach. Such mechanochemical reactions have been previously applied to, for instance, synthesize borohydrides^43^, ^44^, ^45^ or fluoride hydrides.^46^ The successful synthesis was confirmed by a combination of different methods, such as ^1^H MAS (magic angle spinning) NMR spectroscopy and vibrational spectroscopy, together with quantum chemical calculations of the corresponding spectra as well as X‐ray and neutron powder diffraction. Finally, photoluminescence properties of incorporated Eu^2+^ ions in the compound were also studied and a promising orange‐red broad band emission was observed, which shows the typical features of the 4f^6^5d–4f^7^ transition of Eu^2+^.

## Results and Discussion

### Synthesis and crystal structure

**Figure 1:**
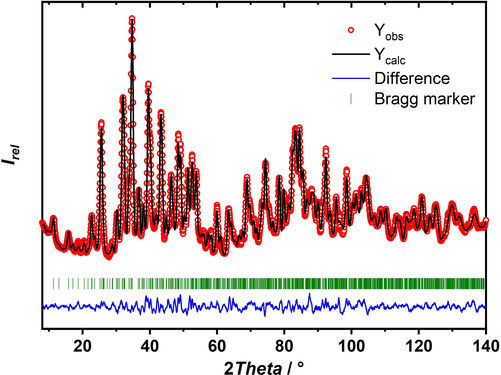
Rietveld refinement of the structural model for the neutron diffraction data of Sr_5_(^11^BO_3_)_3_D measured at the D2B (ILL). *R* values not corrected for background: *R*_p_ 2.09 %, *R*_wp_ 2.67 %, *R*_exp_ 2.59 %, *χ* ^2^ 1.06. Conventional *R* values: *R*_p_ 7.28 %, *R*_wp_ 8.23 %, *R*_exp_ 8.00 %, *χ* ^2^ 1.06. *R*_B_: 4.09 %, *R*_f*‐*factor_: 2.37 %.

Mechanochemical reaction of carefully dried Sr_3_(BO_3_)_2_ with SrH_2_ first yields (after one ′set′ of high‐energy ball milling—a ′set′ consisted of 60 ′cycles′, a ′cycle′ of 2 minutes of milling and 3 minutes pauses; for details of the milling procedure, see the Experimental Section) a powder showing a diffraction pattern with broad reflections of the reactants together with a new set of reflections, which is assigned to the desired compound Sr_5_(BO_3_)_3_H crystallizing in the same structure type as the previously reported Sr_5_(BO_3_)_3_F.^42^ After three more ′sets′ of 60 milling ′cycles′, the reflections belonging to the starting material have disappeared and only the reflections fitting the model of a borate hydride remained. The product synthesized by mechanochemical methods is clearly characterized by low crystallinity, which was improved by heating the sample to 420 °C. In X‐ray diffraction, the atomic form factors of F and O are quite similar and significantly different from the scattering power of H. To distinguish between the desired borate hydride and a possible unwanted borate hydroxide, the simulated X‐ray powder patterns (Cu_Kα_ radiation) of the borate hydride and a so far experimentally unknown borate hydroxide were compared and significant differences in the patterns are visible (Figure S4 in the Supporting Information). As H is a weak X‐ray scatterer, additional neutron powder diffraction data on the high‐resolution neutron powder diffractometer D2b at the ILL, Grenoble, were recorded. Owing to the large absorption of neutrons by ^10^B in the natural isotope mixture ^nat^B, ^11^B‐enriched samples had to be prepared. Additionally, ^1^H shows a high incoherent scattering cross section. Thus, ^2^H‐enriched samples were studied.^47^ A refinement of the structural model for the neutron powder diffraction data of Sr(^11^BO_3_)_3_D is shown in Figure 1 and structural parameters can be found in Tables S1 and S2 in the Supporting Information. The structural model is based on the fluoride analog Sr_5_(BO_3_)_3_F.^42^

The refinements of the structural model for both the X‐ray and neutron powder data of Sr_5_(^11^BO_3_)_3_D, respectively, were found to be in good agreement with the structural model of Sr_5_(BO_3_)_3_F.^42^ The borate deuteride crystallizes orthorhombically in the space group *Pnma* (No. 62) with *a=*7.1982(3) Å, *b=*14.1461(7) Å, *c=*9.8215(4) Å, and *V=*1000.10(8) Å^3^. These obtained lattice parameters are slightly larger than for the fluoride analog (*a=*7.22 Å, *b=*14.09 Å, *c=*9.81 Å, and *V=*998.1 Å^3^),^42^ a usual observation when studying fluorides and hydrides crystallizing in the same structure type, for example, see Ref. 16. The metal–hydride interatomic distances are found to be about 2.42 Å and 2.55 Å, which lie in the typical range of inorganic metal hydrides.^14^, ^48^, ^49^ An additional refinement of the deuterium occupancy at the 4*c* site resulted in a value of 0.915(14). Because the compound is a typical ionic compound, a partial occupation can be excluded. As the occupancy of the deuterium site strongly correlates with the thermal displacement parameter *B*_iso_ (64 %) of the same atom, we assume a full occupation of the deuterium site, resulting in the chemical formula Sr_5_(^11^BO_3_)_3_D. We therefore present both results of the refinement in Tables S1 and S2.

To further distinguish the borate hydride from a potential hydroxide, we also used the structural model we had obtained in quantum chemical calculations for a hypothetical hydroxide and refined that model against the powder neutron pattern of Sr_5_(^11^BO_3_)_3_D. The refinement plot is depicted in the Supporting Information in Figure S5. This structural model yields significantly higher residual values compared with the refinement of the structural model of Sr_5_(BO_3_)_3_F. Hence, based on crystallographic evaluation, we exclude the borate hydroxide as a potential product in our synthesis.

**Figure 2:**
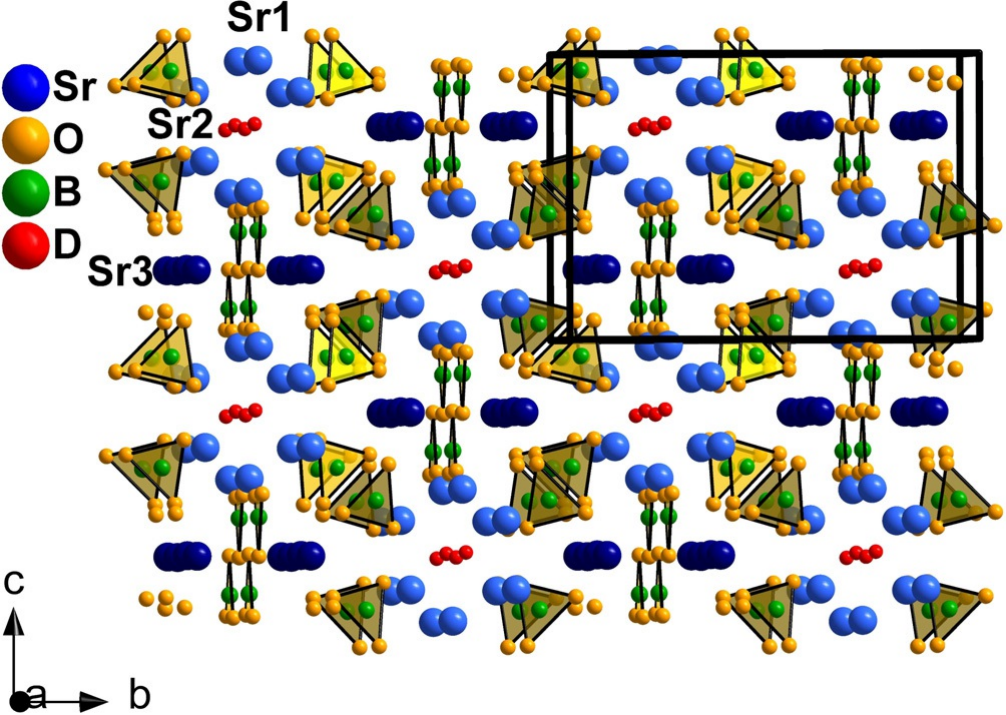
Slightly skewed view along the *a* axis of the crystal structure of Sr_5_(BO_3_)_3_D. Light‐blue (Sr1 and Sr2) and dark‐blue (Sr3) spheres represent strontium atoms, oxygen atoms are drawn in orange, boron atoms in green, and hydrogen in red. The shown radii do not represent the actual size. Other representations can be found in Figure S1 (in the Supporting Information). Coordination spheres of strontium are shown in Figure S2 (in the Supporting Information).

The crystal structure of Sr_5_(BO_3_)_3_H is depicted in Figure 2. The unit cell consists of two distinctive units that run along the *a* axis: Firstly, a double chain of Sr3 centered monocapped square antiprisms (nine oxide anions) and, secondly, channels of hydride surrounded by Sr1 and Sr2 polyhedra. The Sr1 atoms are eight‐fold coordinated by seven oxide anions and one hydride anion. They share faces with two further Sr2 centered polyhedra. The Sr2 polyhedron is composed of six oxide anions and one hydride anion. The different coordination spheres are shown in Figure S2 in the Supporting Information.

The compound decomposes only slowly if stored in air as is evident from X‐ray powder diffraction and vibrational spectroscopy. X‐ray powder patterns recorded after several days of exposure to dry air still revealed the presence of significant amounts of Sr_5_(^11^BO_3_)_3_D or Sr_5_(BO_3_)_3_H (see Figure S7 in the Supporting Information). When Sr_5_(BO_3_)_3_H or Sr_5_(^11^BO_3_)_3_D are added to water, H_2_ or HD gas evolution is observed, respectively. The main decomposition product upon contact with water can be identified as strontium boron hydroxide Sr[B(OH)_4_]_2_ (see Figure S8 in the Supporting Information). It is noteworthy that the gas evolution is still observable after exposure of Sr_5_(^11^BO_3_)_3_D or Sr_5_(BO_3_)_3_H to dry air for several days and subsequent contact with water. Furthermore, elemental analysis on Sr_5_(^11^BO_3_)_3_D resulted in a deuterium content of 0.32 wt % (theoretical value 0.26 wt %).

### ^1^H NMR spectroscopy

**Figure 3:**
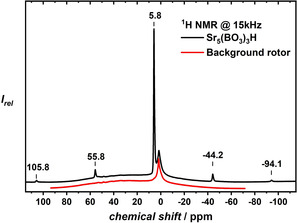
^1^H solid‐state NMR of Sr_5_(BO_3_)_3_H in a 4 mm ZrO_2_ rotor measured with a Bruker AV300. The red curve shows the chemical shift of ^1^H in the blank rotor with Kel‐F cap, which can also be observed in the measurement of the borate hydride (black curve). Sr_5_(BO_3_)_3_H shows a ^1^H chemical shift of 5.8 ppm.

Lately, ^1^H MAS NMR has proven to be a very useful tool to identify hydride species in mixed anionic hydride compounds and differentiate between hydride and hydroxide species.^14^, ^20^, ^50^ In Figure 3, the ^1^H MAS NMR spectrum of Sr_5_(BO_3_)_3_H measured at a spinning frequency of 15 kHz is depicted. At a chemical shift of 5.8 ppm, one sharp signal with additional spinning side bands with a distance of 15 kHz can be observed. Quantum chemical calculations of the solid‐state ^1^H NMR shifts at the DFT‐PBE/USPP level of theory yields a chemical shift of 5.9 ppm, which is in perfect agreement with the observed experimental value. The observation of one sharp signal is also in good agreement with the fact that the structure model features a single site for the hydride ions, which are octahedrally surrounded by Sr^2+^ ions only.

Furthermore, the observed chemical shift is in very good agreement with recent findings on a phosphate hydride (^1^H chemical shift of 5.5 ppm)^14^ and is also in the range of chemical shifts observed in other investigations on metal hydrides.^51^, ^52^, ^53^ As the corresponding borate hydroxide is unknown, we have calculated the ^1^H NMR shift of such a hypothetical Sr_5_(BO_3_)_3_OH at the DFT‐PBE/USPP level of theory. The correspondingly expected chemical shift was at 3.9 ppm, clearly different from the chemical shift found for the hydride. For comparison, in phosphate hydroxide of the apatite family, the ^1^H chemical shift of the OH group is usually located at approximately −0.40 ppm.^14^, ^54^, ^55^

### Vibrational spectroscopy

**Figure 4:**
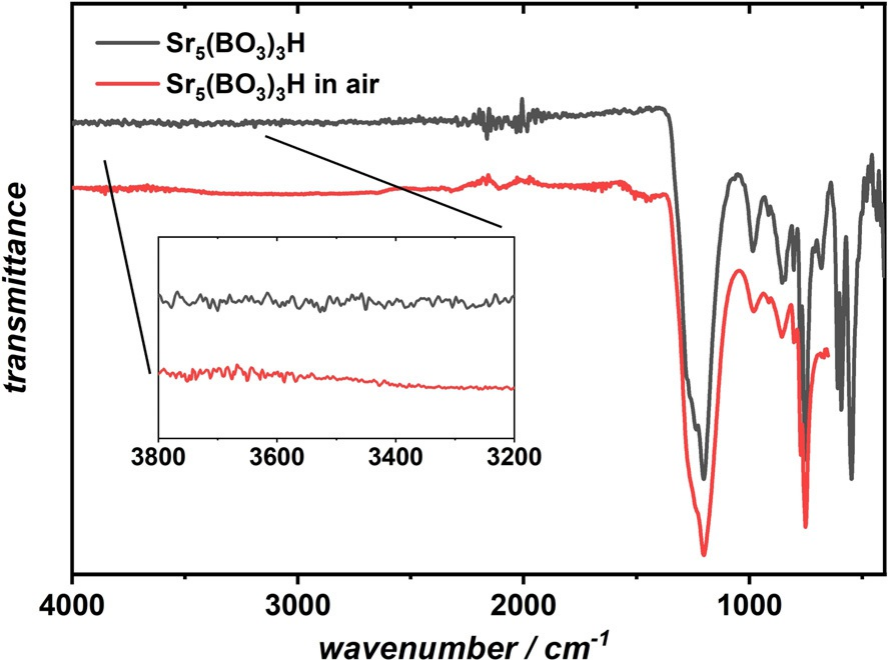
Comparison of the FTIR spectra of Sr_5_(BO_3_)_3_H after synthesis (gray curve) and under ambient (air) conditions (red curve). The hydride vibrations at 990 and 880 cm^−1^ are still visible after exposure to dry air. The signals around 2300 cm^−1^ may either be attributed to the resonance frequencies of the diamond crystal of the device or may stem from the asymmetric stretching vibration of CO_2_ from the atmosphere.

Both the hydride Sr_5_(BO_3_)_3_H as well as the deuteride Sr_5_(^11^BO_3_)_3_D were characterized by vibrational spectroscopy and compared with computational spectra. Figure 4 shows the region between 4000–400 cm^−1^ for the borate hydride as synthesized and after exposure to dry air. The inset depicts the region between 3800–3200 cm^−1^. Usually, stretching vibrations of hydroxide are observed at about 3500–3600 cm^−1^.^56^, ^57^ The absence of such hydroxide‐related bands confirms the assumption that a hydroxide‐free borate hydride has been obtained. Contact with water leads to decomposition and a broad band in the OH region in the FTIR spectra appears (see Figure S11 in the Supporting Information).

**Figure 5:**
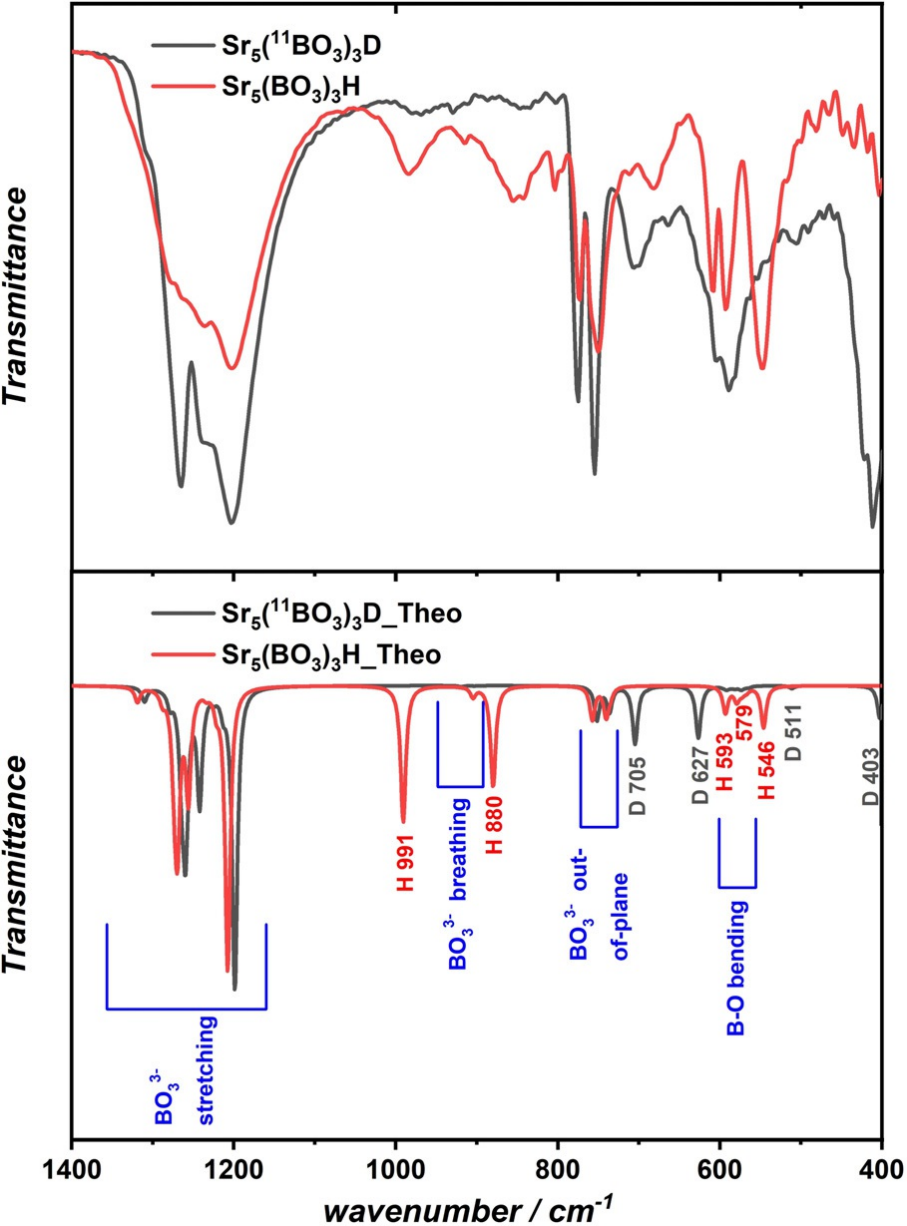
Comparison of the experimental FTIR (top part) and the computational (DFT‐PBE0) IR spectra (lower part) of the hydride Sr_5_(BO_3_)_3_H and the deuteride Sr_5_(^11^BO_3_)_3_D in the spectral range 1400–400 cm^−1^.

In Figure 5, the experimental FTIR as well as the calculated IR spectra of both compounds are shown in the spectral range 1400–400 cm^−1^. The IR‐active vibrations have been assigned by means of quantum chemical calculations and can be found in the lower part of Figure 5 and in Table S5 (in the Supporting Information). In Figure 5, the following color code is applied. The assignment of vibrations belonging to borate groups (i.e., breathing, stretching, bending modes, etc.) are colored in blue, hydride modes in red, and gray is used for deuteride vibrations. For the description of the vibrations belonging to the borate groups, the reader is referred to the Supporting Information. Hydride‐involving modes, the resonances of which are labeled in red, can be found at 991 and 880 cm^−1^. Both modes represent a vibration that is parallel to the plane formed by the three nearest strontium neighbors, herein called ′hydride in‐plane mode′. For a visual representation of these modes and the geometry, see Figure S18 (in the Supporting Information). At 593 cm^−1^ and 546 cm^−1^, sharp ′hydride out‐of‐plane modes′ are observed, which can be described as the hydride vibration that moves perpendicular to the Sr triangle. For comparison, vibrational spectra of Sr_5_(^11^BO_3_)_3_D were also recorded. However, the isotope effect of the B atoms on the IR spectra is almost negligible.

The borate vibrations are only slightly shifted, accounting for the pure ^11^B isotope in the Sr_5_(^11^BO_3_)_3_D compound. The deuteride vibrations, however, can be expected to be shifted to lower wavenumbers by approximately a factor of $\sqrt{2}$ and, indeed, the calculated and experimental IR vibrations of the deuteride are in very good agreement. The ′in‐plane′ modes can now be found at 705 and 627 cm^−1^, respectively, corresponding almost perfectly to a factor of $\sqrt{2}\;$ compared with the hydride vibrations. Although the mode at 705 cm^−1^ is clearly observed in the experimental spectra, the mode at 627 overlaps with B−O bending modes. The ′out‐of‐plane′ modes of the deuteride (=593 cm^−1^ for the hydride) are shifted to the low energetic detection limit of the employed device such that only one of those modes located at around 405 cm^−1^ could be recorded experimentally.

**Figure 6:**
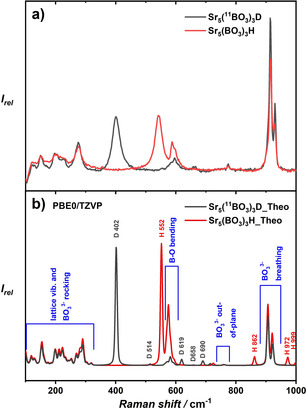
(a) Raman spectra of Sr_5_(BO_3_)_3_H and Sr_5_(^11^BO_3_)_3_D excited with a 532 nm laser. (b) Raman spectra calculated at the DFT‐PBE0/TZVP level of theory.

The experimental and calculated Raman spectra of Sr_5_(BO_3_)_3_H and Sr_5_(^11^BO_3_)_3_D are depicted in Figure 6 and the assignment of vibrations can be found in Table S6 (in the Supporting Information). The color code is equivalent to the IR spectra before. At 552 cm^−1^, a strong signal in the Raman spectrum of the borate hydride can be attributed to the ′out‐of‐plane mode′ respective to the plane formed by the three nearest strontium atoms. Calculated resonances of the hydride modes at 862, 972, and 999 cm^−1^ are related to weak transition intensities, as is observed in the experimental spectra.

The deuteride ′out‐of‐plane vibration′, labeled in gray, is observed at 402 cm^−1^, again in excellent agreement with quantum chemical predictions. Its vibrational energy is also reduced by a factor of $\sqrt{2}$ compared with the respective vibrational transition in the hydride. In addition, the ′in‐plane mode′ is located at 658 cm^−1^. The general assignment of the different B−O modes is also in very good agreement with the collection of vibrational energies found in borates reported in Ref. 57.

### Electronic properties

Additionally, band structures and densities of states for Sr_5_(BO_3_)_3_H and Sr_5_(BO_3_)_3_F have been determined at the DFT‐PBE0/TZVP level of theory (see Figure S20 in the Supporting Information). Both compounds are insulators with an indirect band gap of approximately 7.5 eV for the fluoride compound and 7.0 eV for Sr_5_(BO_3_)_3_H. For band gaps as large as 7 eV, the hybrid PBE0 functional with 25 % of exact exchange may slightly underestimate the experimental gap, whereas the PBE functional is expected to underestimate the band gap even more (here DFT‐PBE predicts a band gap of 4.8 eV for Sr_5_(BO_3_)_3_H).^58^ Owing to the electronegative character of fluoride, the highest valence bands in Sr_5_(BO_3_)_3_F are the oxygen 2p orbitals, which appear as rather flat bands (Figure S20 in the Supporting Information). In contrast, the situation changes in Sr_5_(BO_3_)_3_H. Here, the topmost valence bands are steeper compared with the fluoride analog and the DOS analysis shows that the 1s hydride orbital is mostly responsible for the raising of the valence band and therefore the reduction of the band gap compared with Sr_5_(BO_3_)_3_F. Both compounds can be regarded as ionic materials, but in Sr_5_(BO_3_)_3_H a more covalent behavior owing to the polarizability of hydride is noticeable in the topology of bands. This reduction of the band gap by hydride ligands compared with their fluoride counterparts is in line with previous literature reports and reflects the strong covalent character of the hydride compound.^20^, ^21^

### Luminescence spectroscopy

**Figure 7:**
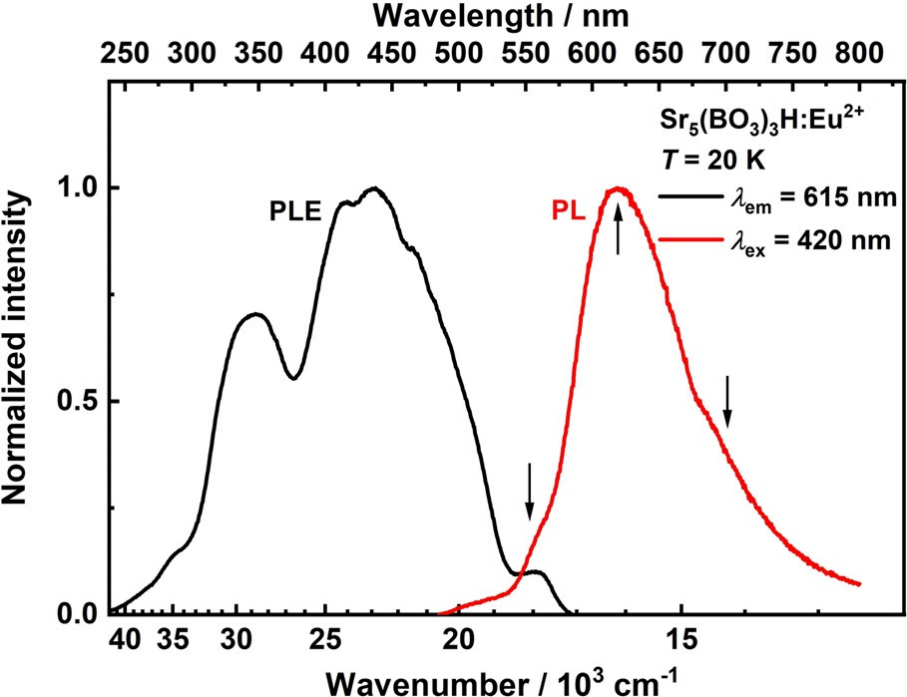
Photoluminescence excitation (PLE) and photoluminescence (PL) of Sr_5_(BO_3_)_3_H:Eu^2+^ at 20 K. Two broad excitation bands are located at 337 nm and 420 nm. At 20 K, the emission band has a maximum at 615 nm and shows two shoulders at around 570 and 690 nm.

Sr_5_(BO_3_)_3_H doped with 3.75 mol % Eu shows orange luminescence upon excitation with violet light (*λ*_ex_=420 nm). Photoluminescence emission (PL) and excitation spectra (PLE) at 20 K are depicted in Figure 7. At 20 K, a broad emission band with a maximum of 615 nm and two additional shoulders at around 570 nm and 690 nm can be observed. The excitation spectrum reveals two broad bands with their respective maxima at 337 nm and 420 nm, which are assigned to the 4f^6^5d^1^–4f^7^ transition of Eu^2+^.

**Figure 8:**
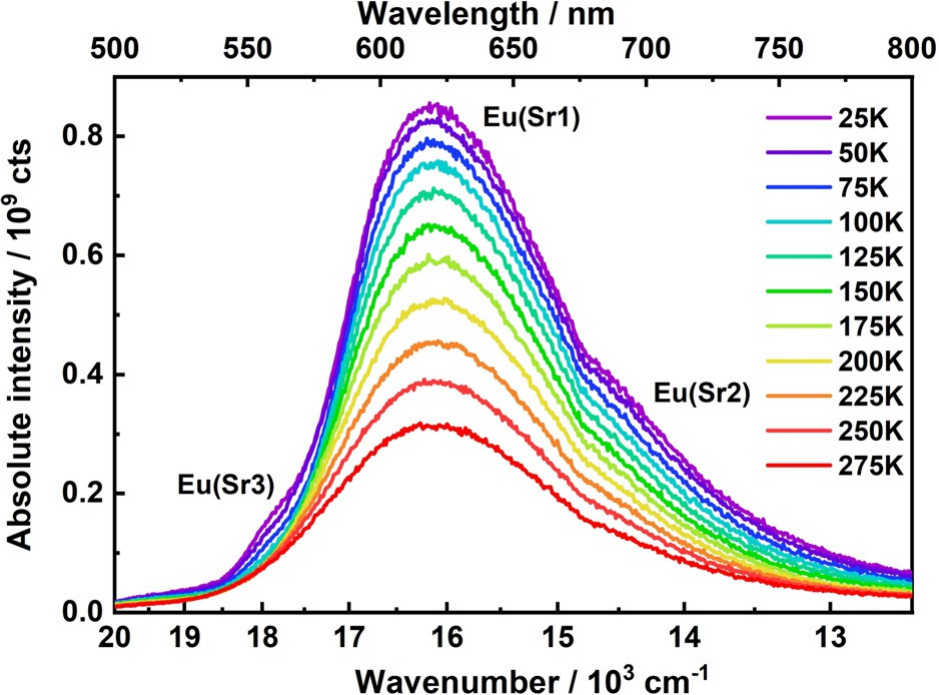
Temperature‐dependent photoluminescence emission spectra of Sr_5_(BO_3_)_3_H:Eu^2+^. The emission maximum and the two shoulders are assigned to the three crystallographically unique strontium sites. With increasing temperature, the shoulder at higher energy vanishes.

Upon increase of the temperature, the absolute intensity of the Eu^2+^‐related emission in the doped borate hydride decreases approximately linearly, which is depicted in Figure 8. In Sr_5_(BO_3_)_3_H, there are three crystallographically unique Sr^2+^ sites, which can be substituted by Eu^2+^ ions. Two of these three sites have H^−^ ions in their coordination sphere. According to the coordination number and the average metal–ligand distance, the crystal field splitting for short metal–ligand distances is expected to be the largest. The Sr3 site has a coordination number of nine with only oxide ions and an average Sr−O distance of *d*(Sr‐O)=2.70 Å (from powder neutron diffraction data, see Table S4 in the Supporting Information). Sr1 is eightfold coordinated with seven oxide ions and one hydride ion. The average Sr–ligand distance is 2.64 Å in that case. Lastly, the Sr2 site has seven anions in its coordination sphere, out of which six are oxide ions and one is a hydride ion. The Sr–ligand distance at that site is about 2.61 Å. Therefore, we tentatively assign the high energy band at around 570 nm to Eu^2+^ ions on the Sr3 site with the longest average Sr–ligand distances and solely more ionic oxide ligands in the first coordination sphere. The lower energetic emission bands are assigned to Eu^2+^ ions on Sr sites with more covalent chemical bonding character to the surrounding ligands owing to the presence of the hydride ion in the coordination sphere (see section on electronic properties). The dominant emission band is tentatively assigned to Eu^2+^ ions on the Sr1 site given the longer average Sr–ligand distances and the low energy shoulder to the sevenfold coordinated Sr2 site with shorter Sr–ligand distances. Actual deconvolution of the photoluminescence emission at 20 K reproduces these three peaks only to a certain degree, because the overlap of the bands from different sites is rather large. The integrated intensity is reduced to half of its initial maximum at around *T*_50 %_=240 K, which is thus estimated as a measure for the quenching temperature (Figure S16 in the Supporting Information).

Normalized temperature‐dependent luminescence spectra are shown in Figure S15 (in the Supporting Information). At 20 K, the full width at half maximum (FWHM) is about 2320 cm^−1^, which decreases slightly until 125 K to about 2250 cm^−1^ and may be attributed to the high energy shoulder vanishing (see Figure S17 in the Supporting Information). This indicates that Eu^2+^ on the Sr3 has a low (=100 K) quenching temperature, which could be related to the higher energy position of the emitting 4f^6^5d state and thermally activated photoionization to the conduction band. From 125 K, the FWHM increases linearly with a larger slope to about 2600 cm^−1^ at room temperature. This increase is usual for broad‐band emitters and related to the thermal excitation of phonon modes, which increase the probability for Stokes‐ and anti‐Stokes vibronic transitions, thus effectively increasing the FWHM of the respective emission band.

**Figure 9:**
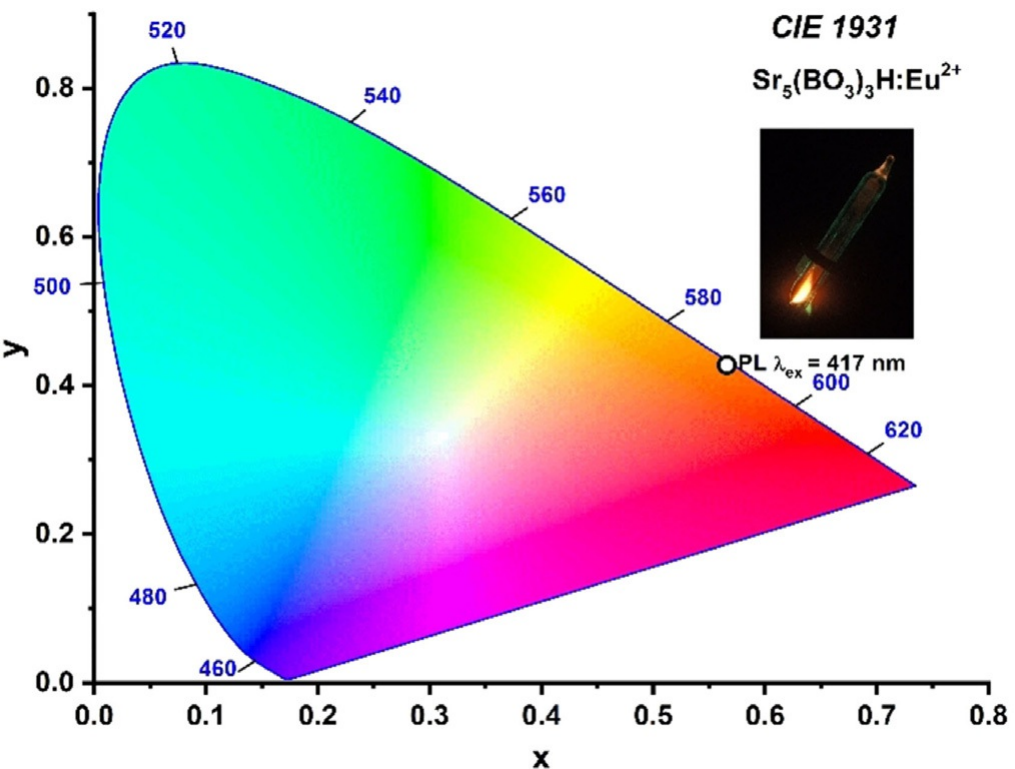
CIE 1931 diagram for orange‐emitting Sr_5_(BO_3_)_3_H:Eu^2+^. The coordinates are *x=*0.56585 and *y=*0.42780.

For phosphors, the emitted color perceived by the human eye is another important material property, which is typically evaluated by the CIE coordinates (Commission Internationale De L′Eclairage). A corresponding plot is depicted in Figure 9. The inset shows the borate hydride in a glass ampoule, which was photographed through UV safety glasses to filter out the violet excitation light. The coordinates according to the CIE diagram are *x=*0.56585 and *y=*0.42780.

Generally, in Eu^2+^‐doped strontium borates, the emission energy was reported to range from UV to orange. Table 1 lists several different strontium borates found in the literature, with their respective maxima of the observable 4f^6^5d^1^→4f^7^ emission bands, average Sr−O distances, and the coordination number around the oxygen atoms. In particular, the latter is the decisive part that determines the emission energies in these compounds.^59^

**Table 1:** Emission maxima, average cation–anion distance, and the maximal coordination number (CN) of oxygen to strontium atoms. This number determines to a large extent the emission energy of Eu^2+^ in these borate compounds.

Compound	*λ*_em_ [nm]	*d*(M–O) [Å]	CN(Sr,max)	Reference
SrB_4_O_7_	367	2.710	2	60
SrLiB_9_O_15_	385	2.764	2	61
SrAl_2_B_2_O_7_	415	2.578	2	62
Sr_2_B_5_O_9_Cl	423	2.578	2	63
SrB_2_O_4_	448	2.617	3	64
Sr_2_Al_2_B_2_O_8_	529	2.675	3	59
Sr_3_(BO_3_)_2_	585	2.601	3	65
Sr_2_Mg(BO_3_)_2_	590	2.657	3	59
Sr_5_(BO_3_)_3_F	600	2.678	4	33
Sr_5_(BO_3_)_3_H	615	2.669	4	This work

Diaz and Keszler found in their studies on Eu^2+^‐doped strontium and barium borates that a low coordination of oxide ions by alkaline earth metal cations, such as in SrB_4_O_7_, results in higher energetic emission. In borates with oxide ions being coordinated by more alkaline earth metal cations, the emission is always widely redshifted.^59^ With CN≤2, UV emission occurs, whereas for CN>3 blue to orange emission is reported. This behavior was explained by a subtle effect on the covalency effect of the Eu^2+^–oxide bonds in borates or aluminates.^59^, ^66^ In the case of many highly charged B^3+^/Al^3+^ ions around oxide anions, they become rather hard Lewis bases or, alternatively, strongly ionic ligands. More Sr^2+^ cations around an oxide ion effectively increase the optical basicity of the ligand. The oxide ions become better σ‐donating ligands and, consequently, the chemical bond between the cations and the oxide ligand is more covalent. The extreme case is SrO:Eu^2+^ without any B^3+^ or Al^3+^ ions and a 4f^6^5d–4f^7^ emission wavelength of 630 nm.^67^ Additional evidence for that interpretation has been recently given in Eu^III^ phosphates, in which a clear linear correlation between the σ‐donating character of the ligands and their optical basicity could be observed.^68^ Thus, the redshift becomes evident with O atoms surrounded by three or more strontium atoms.^59^ In Sr_5_(BO_3_)_3_F:Eu^2+^ and Sr_5_(BO_3_)_3_H:Eu^2+^, oxygen atoms are surrounded by three and four strontium atoms, which will lead to a higher covalency in both cases and should lead to an expected orange emission irrespective of the second anion. For the polyhedra around the different oxygen atoms, see Figure S3 (in the Supporting Information). In the hydride compound, the emission is additionally redshifted compared with the isotypic fluoride compound. This can be explained by the covalency (demonstrated by the DFT calculations) and the additional nephelauxetic effect of the hydride anions, which has also been observed in other Eu^2+^‐doped metal hydrides.^15^, ^16^, ^17^, ^18^, ^19^, ^20^, ^21^, ^69^, ^70^ Thus, insertion of hydride anions in different mixed anionic compounds allows a fine‐tuning of the emission properties. On the other hand, studying the Eu^2+^ luminescence may also be a tool to indirectly prove the incorporation of hydride anions in new mixed anionic materials, where such incorporation is difficult to detect with other characterization methods.

## Conclusion

To the best of our knowledge, we report the synthesis of the first borate hydride. The current findings could open up a new class of materials. The proof of hydrogen incorporation in the presented compound is far from trivial and could be confirmed by several independent methods such as neutron powder diffraction, solid‐state NMR and vibrational spectroscopy, quantum chemical electronic calculations, and luminescence spectroscopy. Unlike the conventionally experienced instability of hydrides in air, the compounds decompose only slowly in dry air. If doped with Eu^2+^, the compound shows orange‐red luminescence emission upon excitation with violet light. Incorporation of hydride ligands in the coordination environment of Eu^2+^ is shown to be an effective method to tune the Eu^2+^ emission color and induce a redshift to realize the desired emission color.

## Experimental Section

### Synthesis

For the synthesis of the borate hydride Sr_5_(BO_3_)_3_H, a mixture of Sr_3_(BO_3_)_2_ and SrH_2_ (100 % excess) was used. Sr_3_(BO_3_)_2_ can be synthesized by heating stoichiometric amounts of SrCO_3_ (Alfa Aesar, 99 %) and H_3_BO_3_ (Alfa Aesar, 99 %) in a muffle furnace at 1100 °C for 48 h. SrH_2_ was obtained by reacting strontium metal (Alfa Aesar, 99 %) at 60 bar hydrogen pressure (Westfalen, 99.9 %) in a hydrogen resistant autoclave (Böhler L718) at 550 °C for 48 h. Sr_5_(BO_3_)_3_H was then synthesized mechanochemically by mixing dried Sr_3_(BO_3_)_2_ and SrH_2_ in a Fritsch Pulverisette 7 Premium Line ball mill in a 20 mL WC (tungsten carbide) grinding bowl with 10 WC balls (10 mm diameter) at a speed of 600 rpm for a total of 240 cycles (4 times 60 cycles) under argon atmosphere. One cycle consisted of 2 min of milling and 3 min pauses to reduce heating of the sample and the herein called set comprised of 60 cycles. Between each set, the sample was removed from the grinding bowl to crush it again. To improve the crystallinity of the milled sample, it was then heated in an evacuated glass ampoule to 420 °C for 10 days. Deuterated (D_2_ gas by AirLiquide, 99.8 %) and ^11^B‐enriched samples Sr_5_(^11^BO_3_)_3_D for neutron diffraction were synthesized by using SrD_2_ and H_3_^11^BO_3_ (Sigma–Aldrich, isotopic purity ≥99 %, chemical purity ≥98 %) as starting materials following the procedure described above for the hydride. For Eu‐doping, Eu_2_O_3_ was added during the synthesis of Sr_3_(BO_3_)_2_. In previous works, it has been shown that Eu^3+^ is inherently reduced to Eu^2+^ during the synthesis of borates.^71^, ^72^ Especially in our case, the reaction conditions are reductive owing to the additional presence of SrH_2_. All handling was performed under argon atmosphere.

### X‐ray and neutron diffraction

X‐ray diffraction data was collected with a Stoe StadiP diffractometer with transmission geometry, Cu_Kα1_ radiation and a solid‐state detector (Mythen 1 K) in 0.3 mm glass capillaries.

Neutron diffraction experiments were conducted with the high‐resolution two‐axis diffractometer D2B at the Institute Laue‐Langevin (ILL), Grenoble, with a wavelength of 1.594 Å over the course of 5 h. Samples were enclosed in air‐tight 8 mm vanadium cylinders. Rietveld refinement of X‐ray and neutron diffraction data was carried out by using the FullProf program package.^73^ Cell parameters, atomic positions, thermal isotropic displacement *B*_iso_, profile and shape parameters as well as asymmetry functions have been refined. Background points were refined with scalable height. Structural parameters are shown in Tables S1–S3 (in the Supporting Information). The raw data can be requested under https://doi.org/10.5291/ILL‐DATA.EASY‐475.

Deposition Number 1999692 contains the supplementary crystallographic data for this paper. These data are provided free of charge by the joint Cambridge Crystallographic Data Centre and Fachinformationszentrum Karlsruhe Access Structures service www.ccdc.cam.ac.uk/structures.

### Nuclear magnetic resonance

Magic angle spinning (MAS) solid‐state ^1^H NMR spectra were collected with a Bruker AV300 at 7.05 T with a spinning frequency of 15 kHz in 4 mm ZrO_2_ rotors with a Kel‐F cap at room temperature. To determine ^1^H background signals, the empty rotor and cap was measured.

### Quantum chemical calculations

Solid‐state ^1^H NMR chemical shifts of Sr_5_(BO_3_)_3_H and a hypothetical hydroxide Sr_5_(BO_3_)_3_OH were calculated by using the DFT‐PBE method^74^ and GIPAW formalism as implemented in the CASTEP and CASTEP‐NMR program packages.^75^, ^76^, ^77^ Vibrational spectra of Sr_5_(^nat^BO_3_)_3_^1^H as well as Sr_5_(^11^BO_3_)_3_D were calculated with the CRYSTAL program package^78^ at the DFT‐PBE0/TZVP level of theory. Full computational details can be found in the Supporting Information.

### Infrared spectroscopy

FTIR spectra were recorded with a Spectrum Two (UATR TWO, PerkinElmer) spectrometer with a diamond sensor. To avoid water and oxygen contamination, the device was operated inside an argon‐filled glovebox. Measurements were taken between 4000 and 500 cm^−1^ with a spectral resolution of 1 cm^−1^. Additionally, one sample (Figure 5, red curve) was measured in air with an Agilent Cary 630 FTIR spectrometer with a diamond sensor.

### Raman spectroscopy

Raman spectra were recorded from powders sealed in glass capillaries (0.3 mm diameter) with a Renishaw inVia Reflex Raman System equipped with a CCD detector and a *λ*=532 nm laser in the range 100–1000 cm^−1^.

### Luminescence spectroscopy

Room‐temperature photoluminescence and emission were recorded with a Fluorolog FL322 equipped with a 450 W Xenon lamp and double monochromator in the excitation (1200 l mm^−1^, 300 nm blaze) and double monochromator (1200 l mm^−1^, 500 nm blaze) in the emission compartment, respectively. The emitted light was detected with a Hamamatsu‐R928P photomultiplier tube. Samples were enclosed in quartz ampoules with 5 mm diameter. Temperature‐dependent photoluminescence spectra were acquired with an Edinburgh FLS920 spectrometer equipped with a 450 W Xe lamp, 0.25 m double grating monochromators in both excitation (1200 l mm^−1^, 300 nm blaze) and emission (1200 l mm^−1^, 500 nm blaze) compartment and a Hamamatsu‐R928P photomultiplier tube as detection system. The sample was cooled with an Oxford Instruments liquid He flow cryostat with external temperature control unit. All spectra were corrected for the wavelength‐dependent sensitivity of the PMT and monochromator light output, whereas excitation spectra were corrected for the reference signal of the Xe lamp and grating efficiency.

### Elemental analysis

Elemental analysis was conducted with a Vario El microanalyzer.

## Conflict of interest

The authors declare no conflict of interest.

## Supporting information

As a service to our authors and readers, this journal provides supporting information supplied by the authors. Such materials are peer reviewed and may be re‐organized for online delivery, but are not copy‐edited or typeset. Technical support issues arising from supporting information (other than missing files) should be addressed to the authors.

Supplementary
