# Supplementary material for: Borate Hydrides as a New Material Class: Structure, Computational Studies, and Spectroscopic Investigations on Sr5(BO3)3H and Sr5(11BO3)3D
Source: Chemistry. 2020 Aug 17;26(51):11742–50. doi: 10.1002/chem.202002273 (PMC7540042; doi:10.1002/chem.202002273)
Supplement: Supplementary file 1 — Supplementary [file CHEM-26-11742-s001.pdf]

Chemistry–A European Journal

Supporting Information

Borate Hydrides as a New Material Class: Structure, Computational Studies, and Spectroscopic Investigations on $Sr_5(BO_3)_3H$ and $Sr_5(^{11}BO_3)_3D$

Thomas Wylezich,[a, b, c] Renaud Valois,[c, d] Markus Suta,[e] Alexander Mutschke,[c] Clemens Ritter,[f] Andries Meijerink,[e] Antti J. Karttunen,[g] and Nathalie Kunkel*[a, b, c]

Structure analysis

Table S1: Refined lattice parameters and atomic parameters of the structural base model for $Sr_5(^{11}BO_3)_3D$ (*Pnma*) at 300 K obtained from powder neutron diffraction data.

**$Sr_5(^{11}BO_3)_3D$**		*a* = 7.1982(3) Å, *b* = 14.1461(7) Å, *c* = 9.8215(4) Å, *V* = 1000.10(8) Å^3				
Atom	Site	x	y	z	B_{iso}(Å^2)	s.o.f.
Sr3	8*d*	0.0234(5)	0.1108(2)	0.2531(5)	1.25(7)	1
O5	8*d*	0.0928(6)	0.5870(3)	0.1565(6)	1.40(7)	1
B1	8*d*	0.2072(6)	0.5382(3)	0.0607(5)	2.07(8)	1
Sr2	8*d*	0.2486(5)	0.6195(2)	0.3703(3)	0.84(7)	1
O2	8*d*	0.2734(7)	0.0700(3)	0.4342(5)	0.99(8)	1
O3	8*d*	0.2827(7)	0.0450(2)	0.1038(5)	0.75(6)	1
O4	4*c*	0.1184(12)	¼	0.0445(8)	1.87(15)	1
D	4*c*	0.1151(8)	¼	0.7466(8)	2.65(14)	1
O1	4*c*	0.2781(8)	¼	0.2528(5)	0.26(8)	1
B2	4*c*	0.2737(9)	¼	0.1134(6)	1.06(8)	1
Sr1	4*c*	0.2868(9)	¼	0.5191(4)	0.69(8)	1
O6	4*c*	0.4494(11)	¼	0.0428(7)	1.04(13)	1

Table S2: Additional refinement of lattice parameters and atomic parameters of the structural base model for $Sr_5(^{11}BO_3)_3D_{0.92}$ (*Pnma*) at 300 K obtained from refinement of powder neutron diffraction data. Here, the occupancy for deuterium at the Wyckoff 4*c* site is refined.

**$Sr_5(^{11}BO_3)_3D_{0.92}$**		*a* = 7.1981(3) Å, *b* = 14.1462(7) Å, *c* = 9.8218(4) Å, *V* = 1000.11(7) Å^3				
Atom	Site	x	y	z	B_{iso}(Å^2)	s.o.f.
Sr3	8*d*	0.0236(5)	0.1107(2)	0.2533(5)	1.24(6)	1
O5	8*d*	0.0929(6)	0.5873(3)	0.1565(6)	1.38(7)	1
B1	8*d*	0.2071(6)	0.5385(3)	0.0608(5)	2.13(8)	1
Sr2	8*d*	0.2485(5)	0.6197(2)	0.3701(3)	0.85(7)	1
O2	8*d*	0.2734(7)	0.0701(3)	0.4342(5)	0.99(8)	1
O3	8*d*	0.2826(7)	0.0451(2)	0.1034(5)	0.79(6)	1
O4	4*c*	0.1181(11)	¼	0.0442(8)	1.93(15)	1
D	4*c*	0.1154(8)	¼	0.7457(8)	1.95(17)	0.915(14)
O1	4*c*	0.2783(8)	¼	0.2524(5)	0.31(8)	1
B2	4*c*	0.2741(9)	¼	0.1131(6)	1.10(8)	1
Sr1	4*c*	0.2871(9)	¼	0.5196(4)	0.70(8)	1
O6	4*c*	0.4494(10)	¼	0.0422(7)	1.07(12)	1

Table S3. Crystal data and structure refinement of the two models $Sr_5(^{11}BO_3)_3D$ and the hypothetical compound $Sr_5(^{11}BO_3)_3OD$ against the same data set (Rietveld method, powder neutron diffraction data, T = 298 K). The hypothetical structure model $Sr_5(^{11}BO_3)_3OD$ has been found to be invalid.

**Empirical formula**		$Sr_5(^{11}BO_3)_3D$	$Sr_5(^{11}BO_3)_3OD$
Neutron wavelength/ *Å*		1.5940	1.5940
Space group, *Z*		*P n m a* (62)	$P\,2_1\,2_1\,2_1$ (19)
Unit cell dimensions/*Å*			
a =		7.1982(3)	7.1984(9)
b =		14.1461(7)	14.1364(18)
c =		9.8215(4)	9.8141(10)
Cell volume/$Å^3$			
V =		1000.1(8)	998.7(2)
Calculated density/g cm^{-3}		4.10(1)	4.20(1)
Step scan increment 2θ/deg		0.05	0.05
2θ range/deg		2 – 159.5	2 – 159.5
Program for refinement		*FullProf*	*FullProf*
η		0.37(1)	–
Caglioti parameters			
	U =	0.52(2)	0.68(7)
	V =	-0.69(4)	-0.79(10)
	W =	0.62(1)	0.72(3)
Asymmetry 1		0.061(6)	0.351(19)
Asymmetry 2		0.029(3)	0.039(9)
No. of reflections		1073	1205
No. of refined parameters		52	10
R_B		4.1	11.8
R_P		7.3	16.5
R_{exp}		8.0	7.7
R_{wp}		8.2	19.5
χ^2		1.06	6.31

Table S4: Atomic distances for the three strontium polyhedra obtained by the structure refinement of $Sr_5(^{11}BO_3)_3D$.

**M**	**L**	**Number of L**	**d(M-L) / Å**
Sr1	O4	1	2.4673
	D	1	2.5534
	O6	1	2.5036
	O1	1	2.6162
	O2	2	2.6811
	O5	2	2.8087
	Avg.	8	2.6400

**M**	**L**	**Number of L**	**d(M-L) / Å**
Sr2	D	1	2.4180
	O5	1	2.4245
	O5	1	2.5336
	O3	1	2.5339
	O4	1	2.6629
	O2	1	2.7590

**M**	**L**	**Number of L**	**d(M-L) / Å**
Sr3	O3	1	2.4174
	O3	1	2.5496
	O2	1	2.5952
	O1	1	2.6455
	O2	1	2.6373
	O1	1	2.6905
	O4	1	2.9228
	O6	1	2.8600
	O5	1	2.9965
	Avg.	9	2.7016

O6	1	2.8826
Avg.	7	2.6064

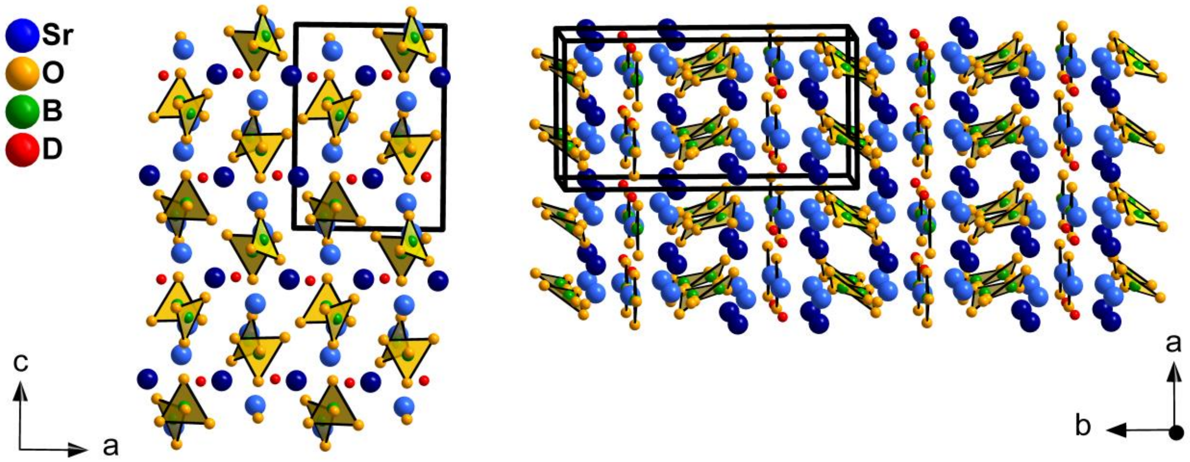

Figure S1: Additional views on the crystal structure of $Sr_5(^{11}BO_3)_3D$. In the left view the hydride/deuteride anions run along the a axis. Right picture shows the view on the c axis.

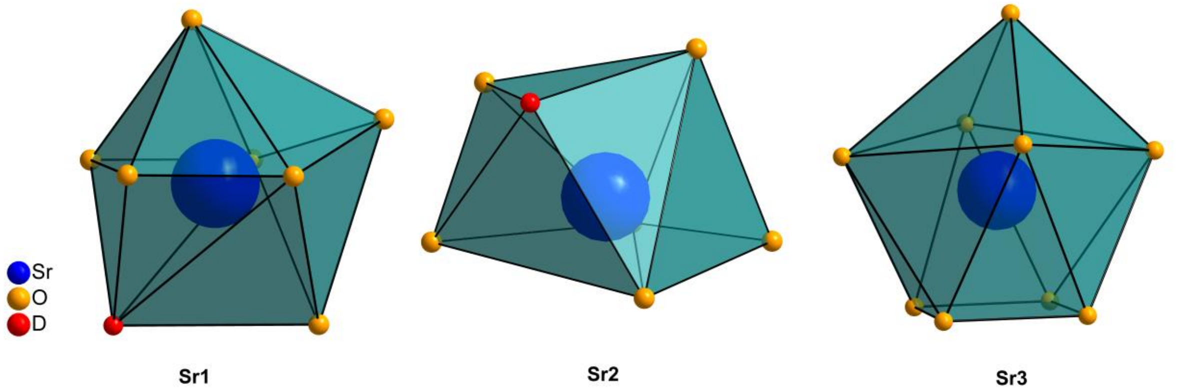

Figure S2: Coordination spheres of the three distinctive Sr-polyhedra. Atomic distances are given in Table S4. Sr1 is eight-fold coordinated by 7 oxygen and 1 hydrogen atom (SrO_7H). The second coordination sphere of strontium (Sr2) comprises of 6 oxide anions and 1 hydride anion (SrO_6H). Sr3 is surrounded by 9 oxide anions and can be described as a slightly distorted monocapped square antiprism that forms a double channel along the a-axis.

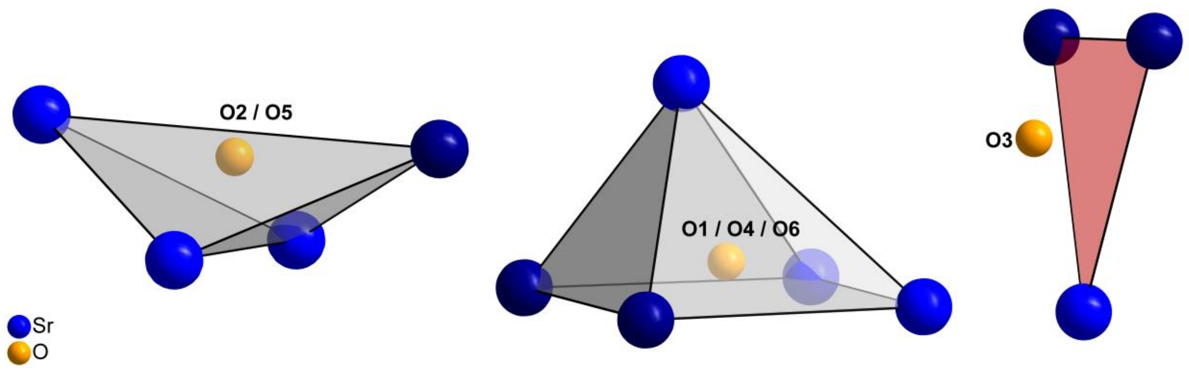

Figure S3: Coordination spheres of oxygen centred polyhedra, that are necessary for understanding the photoluminescence emission spectra.

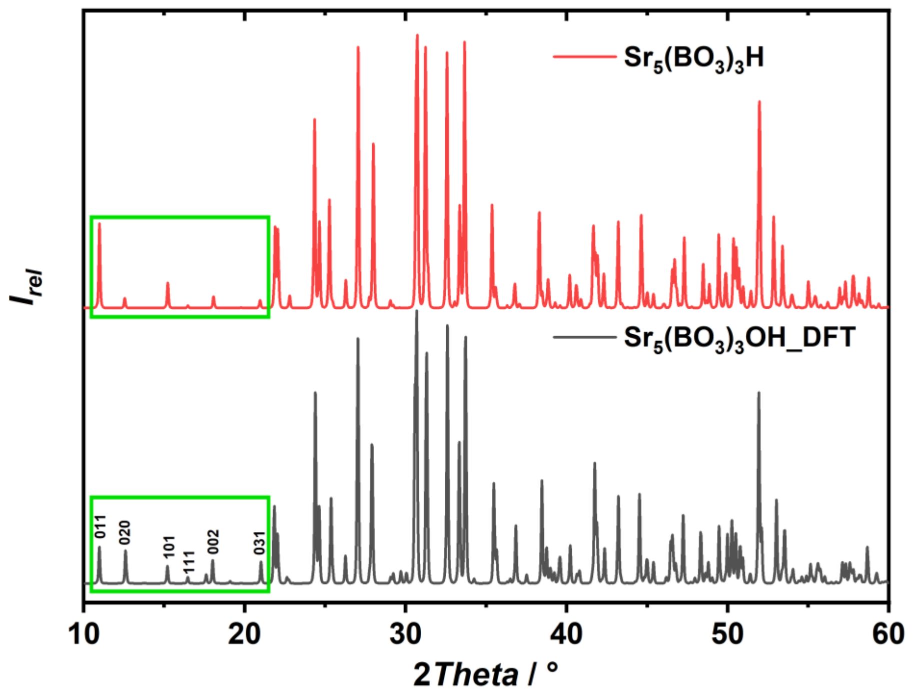

Figure S4: Comparison of the simulated X-ray patterns with $Cu_{K\alpha}$ radiation for the compounds $Sr_5(BO_3)_3X$ (X = OH, H). The hydride pattern differs from the patterns of hydroxide the most at the 011 and 020 reflections. Angular range with significant differences is highlighted in green. The hypothetical crystal structure used for X = OH was obtained from a quantum chemical structure optimization.

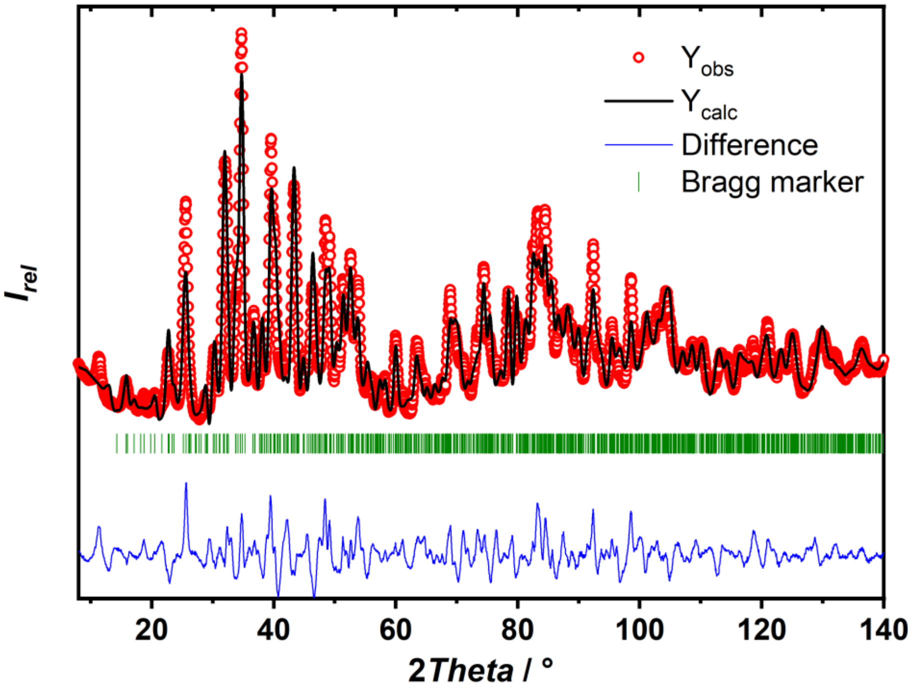

Figure S5: Rietveld refinement of the structural model $Sr_5(^{11}BO_3)_3OD$ obtained by quantum chemical simulation for the powder neutron diffraction data of $Sr_5(^{11}BO_3)_3D$. The refinement is significantly worse compared to the $Sr_5(BO_3)_3F$ structural model. Bragg markers: Structural model $Sr_5(^{11}BO_3)_3OD$. R-values not corrected for background: R_p: 4.98, R_{wp}: 6.57, R_{exp}: 2.61, χ^2: 6.31. Conventional R-values: R_p: 16.5, R_{wp}: 19.5, R_{exp}: 7.76, χ^2: 6.31.

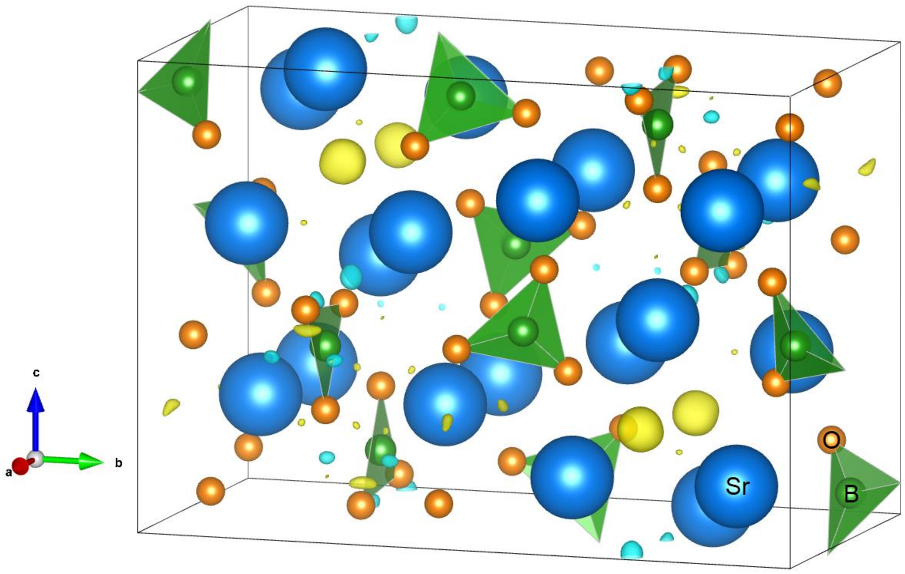

Figure S6: Difference Fourier map of the refinement $Sr_5(^{11}BO_3)_3\square$, in which the deuterium position was kept unoccupied (symbol □, in FullProf *Occ* and B_{iso} were set to 0). Sr is shown in blue, boron in green, and oxygen in orange. The positive, round-shaped residual density (yellow isosurface) is in good agreement with the deuterium position in $Sr_5(^{11}BO_3)_3D$. Graphic representation is shown in VESTA.[1]

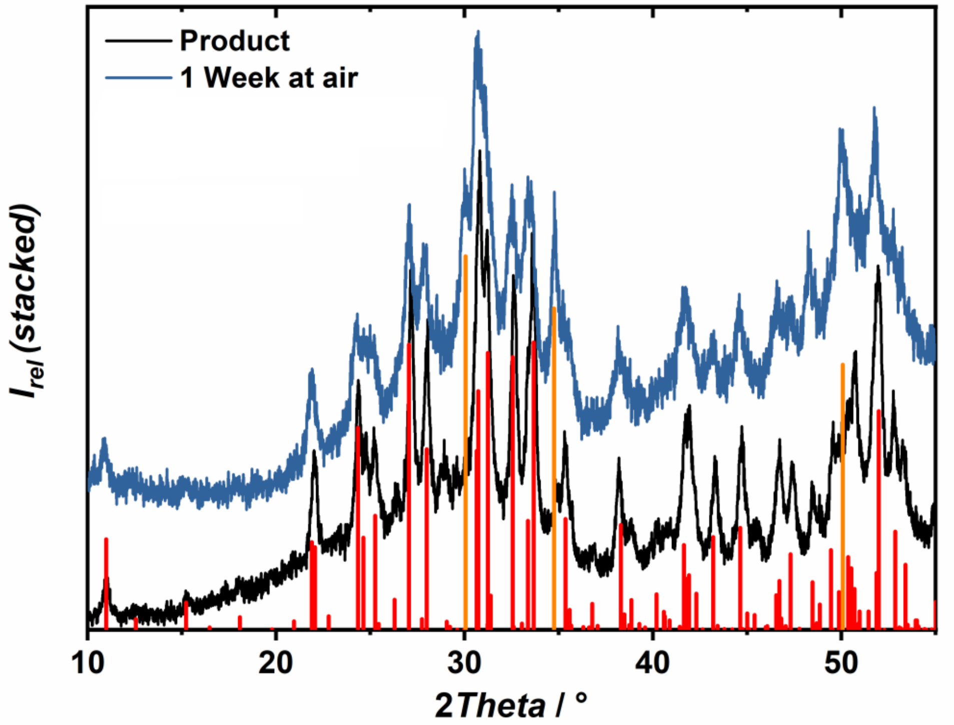

Figure S7: Short scan XRD pattern of $Sr_5(^{11}BO_3)_3D$ after annealing followed by one-week exposure to air. Vertical ticks: Simulated pattern of $Sr_5(BO_3)_3H$ (red), simulated pattern of SrO (orange).

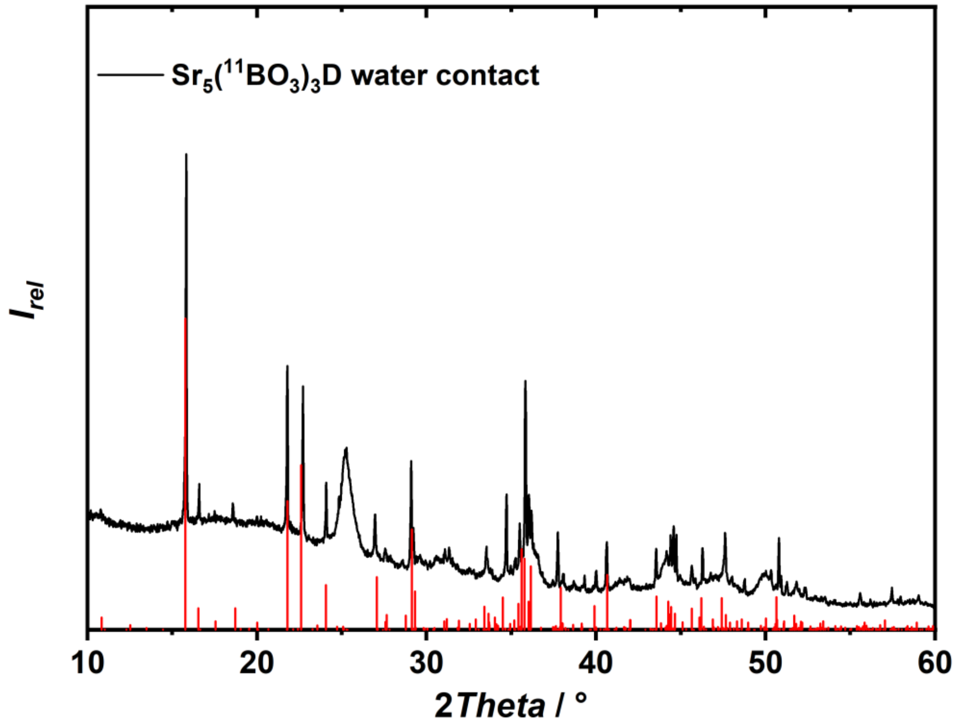

Figure S8: XRD pattern of $Sr_5(^{11}BO_3)_3D$ after pouring water on the sample. Red ticks: Simulated pattern of $Sr[B(OH)_4]_2$.

Spectroscopy

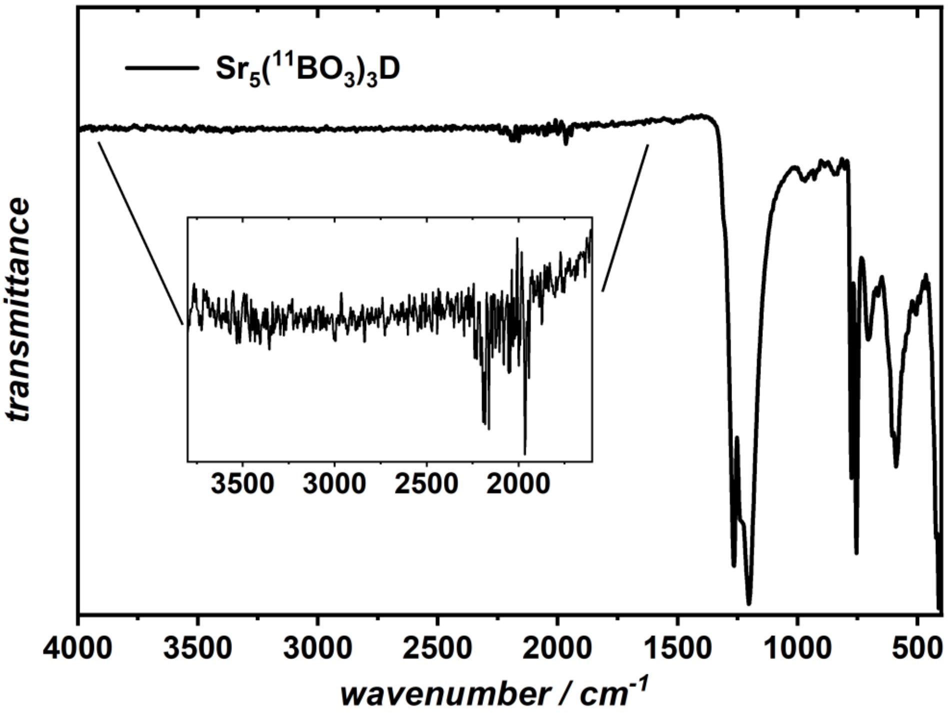

Figure S9: Complete range FT-IR spectrum of $Sr_5(^{11}BO_3)_3D$. At around 2000 cm^{-1} the resonance frequencies of the diamond crystal are visible.

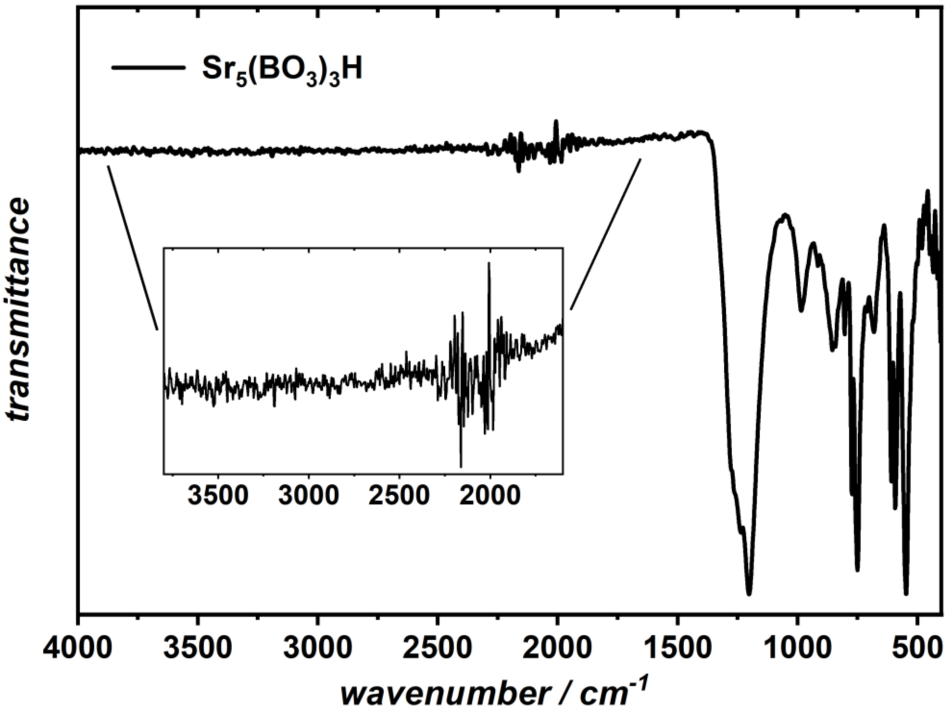

Figure S10: Complete range FT-IR spectrum of $Sr_5(BO_3)_3H$. At around 2000 cm^{-1} the resonance frequencies of the diamond crystal are visible.

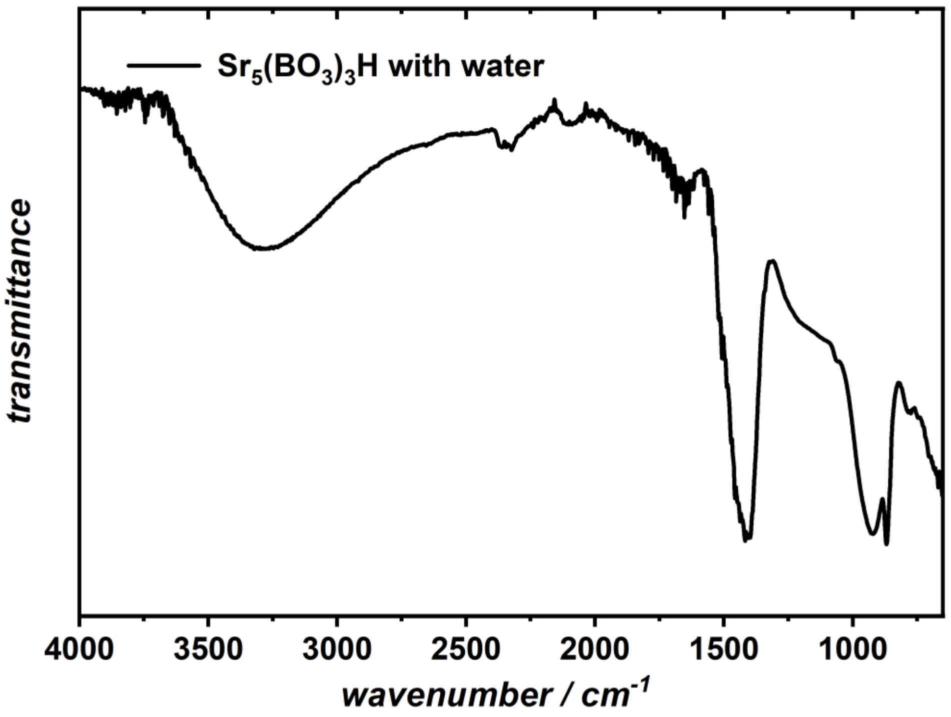

Figure S11: Complete range FT-IR spectrum of $Sr_5(BO_3)_3H$ after pouring water onto the sample.

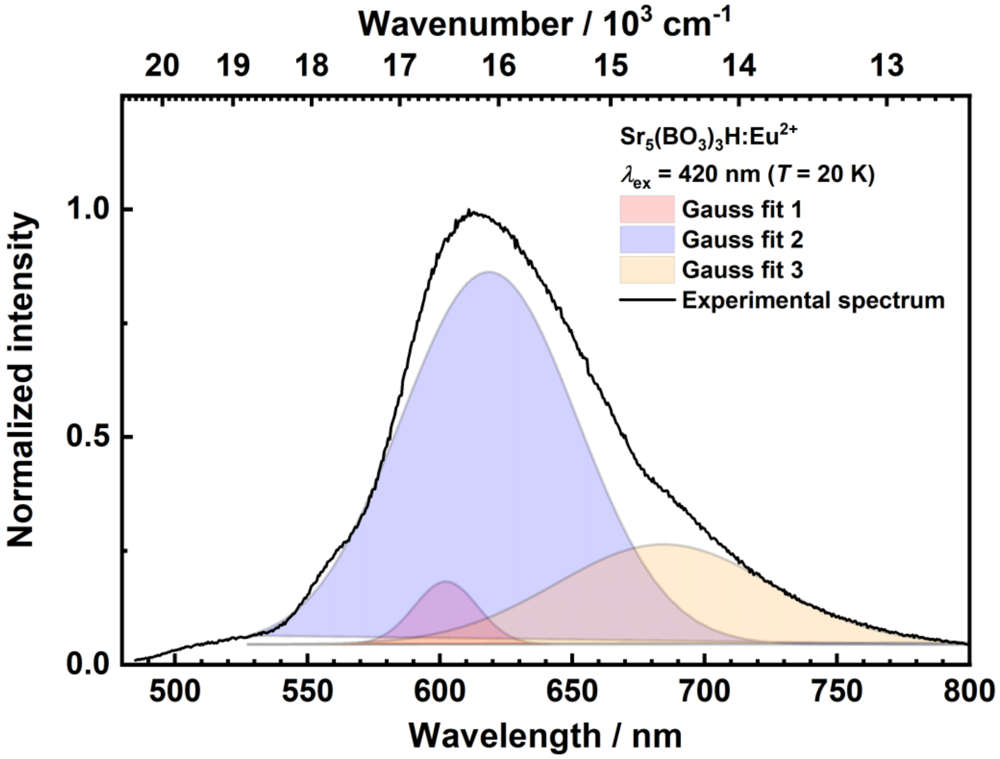

Figure S12: Deconvolution of the emission of $Sr_5(BO_3)_3H:Eu^{2+}$ results in three distinctive peaks.

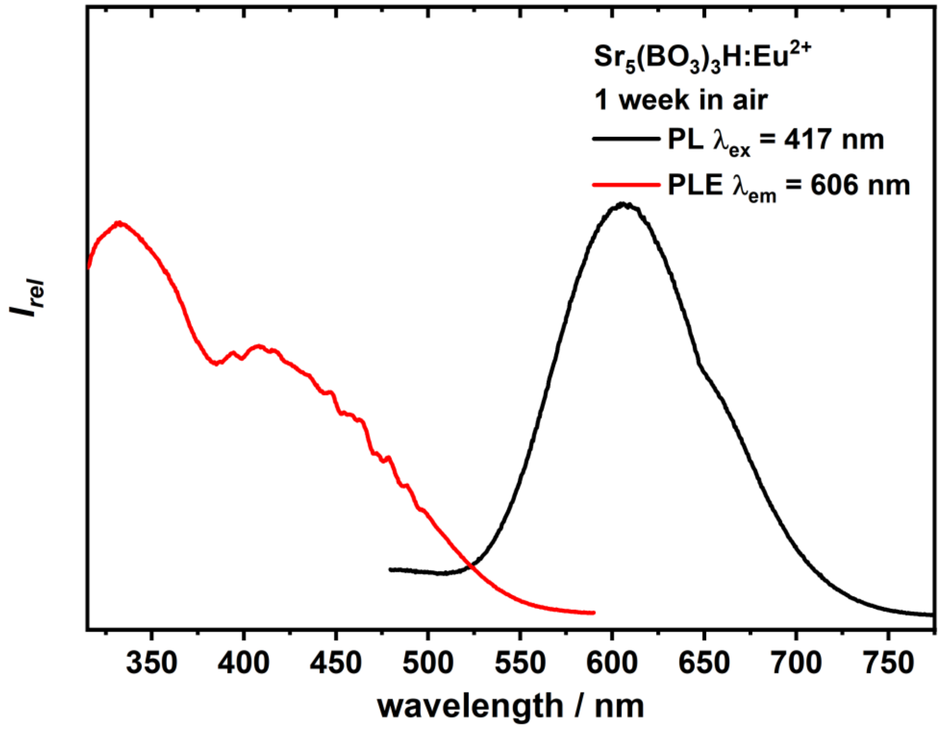

Figure S13: PL and PLE spectra of $Sr_5(BO_3)_3H:Eu^{2+}$ after one week exposure to air.

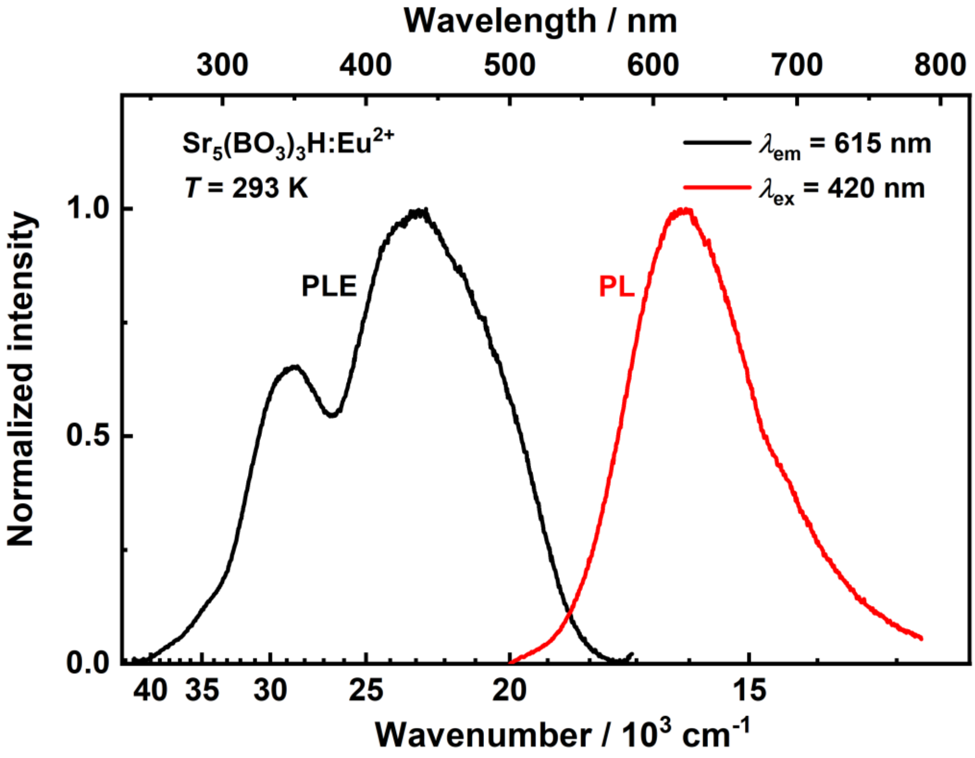

Figure S14: PL and PLE spectra of $Sr_5(BO_3)_3H:Eu^{2+}$ at room temperature.

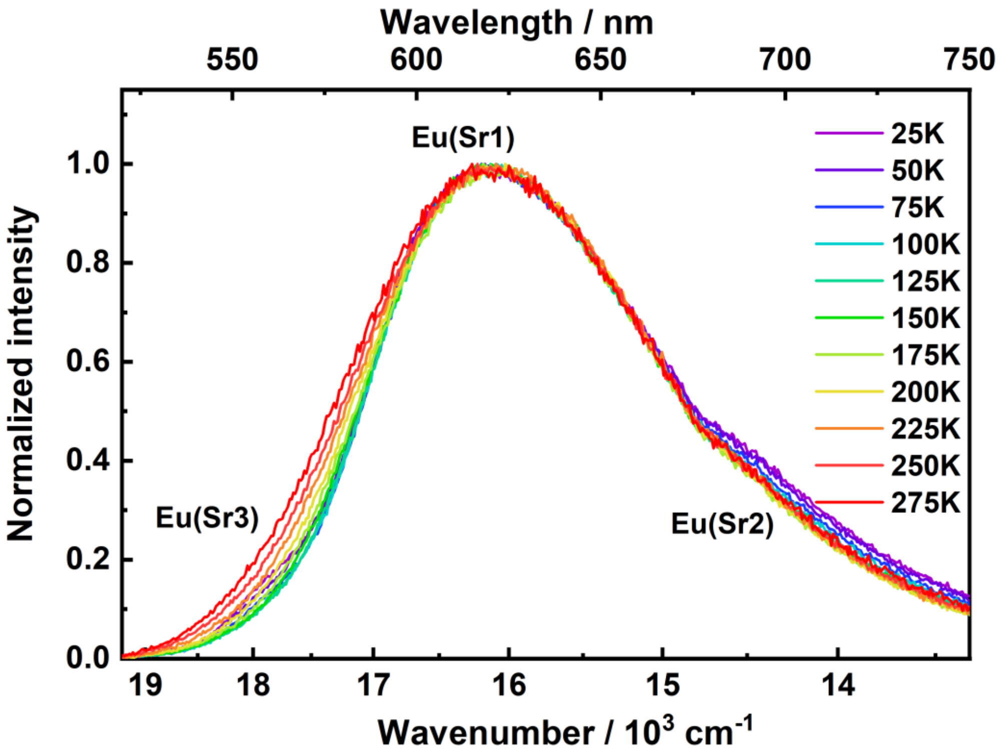

Figure S15: Normalized temperature dependent PL spectra of $Sr_5(BO_3)_3H:Eu^{2+}$.

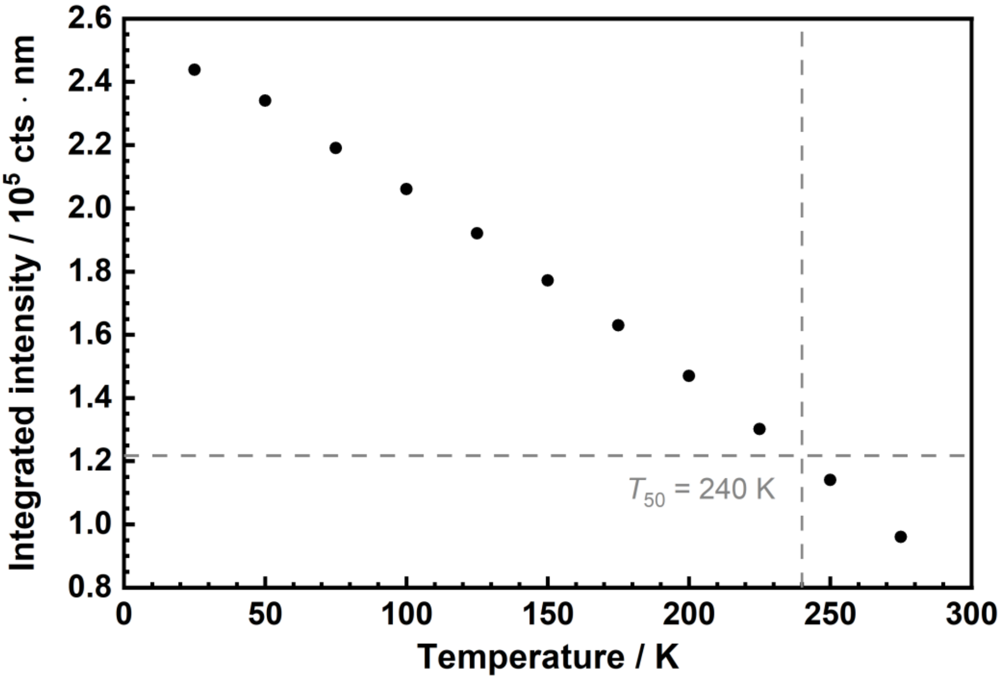

Figure S16: Integrated emission of $Sr_5(BO_3)_3H:Eu^{2+}$ in the temperature range 25 K to 275 K. The quenching temperature $T_{50\%}$ is determined to be approximately 240 K.

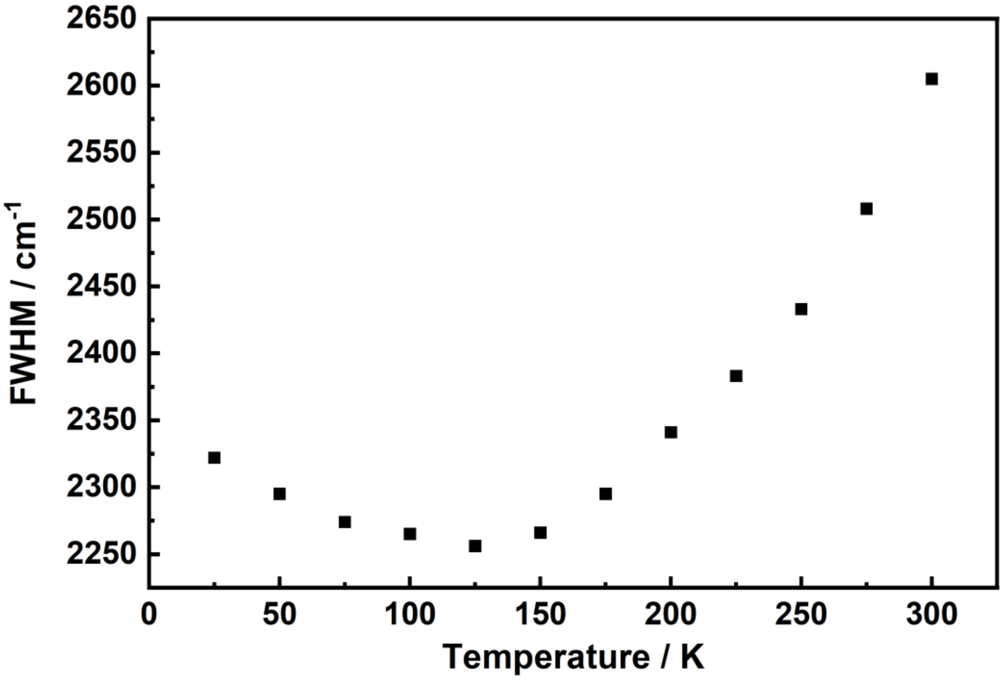

Figure S17: FWHM of the emission of $Sr_5(BO_3)_3H:Eu^{2+}$ in the temperature range 25 K to 300 K.

Table S5: Vibrational IR data obtained from quantum chemical calculations at the DFT-PBE0/TZVP level of theory. The frequency, irreducible representation, intensity, and assignment of the mode are given for $Sr_5(^{nat}BO_3)_3H$ and $Sr_5(^{11}BO_3)_3D$. Vibrations marked with asterisks are illustrated in Figure S18.

$Sr_5(BO_3)_3H$				$Sr_5(^{11}BO_3)_3D$			
Frequency [cm^{-1}]	Γ_{irrep}	Intensity [km/mol]	Assignment	Frequency [cm^{-1}]	Γ_{irrep}	Intensity [km/mol]	Assignment
1319	B_{2u}	581	B-O stretching	1310	B_{2u}	557	B-O stretching
1288	B_{2u}	443	B-O stretching	1279	B_{2u}	568	B-O stretching
1275	B_{1u}	216	B-O stretching	1265	B_{1u}	63	B-O stretching
1273	B_{3u}	2301	B-O stretching	1264	B_{3u}	2676	B-O stretching
1271	B_{1u}	698	B-O stretching	1260	B_{2u}	4915	B-O stretching
1269	B_{2u}	4979	B-O stretching	1258	B_{1u}	1154	B-O stretching
1256	B_{3u}	4169	B-O stretching	1242	B_{3u}	4398	B-O stretching
1234	B_{1u}	179	B-O stretching	1225	B_{1u}	209	B-O stretching
1221	B_{2u}	667	B-O stretching	1213	B_{2u}	655	B-O stretching
1208	B_{1u}	10964	B-O stretching	1199	B_{1u}	11726	B-O stretching
991	B_{1u}	5288	'Hydride in plane stretching'*	924	B_{2u}	0	BO_3^{3-} breathing
973	B_{2u}	4	'Hydride in plane stretching'*	920	B_{1u}	48	BO_3^{3-} breathing
922	B_{2u}	3	BO_3^{3-} breathing	903	B_{1u}	6	BO_3^{3-} breathing
919	B_{1u}	1	BO_3^{3-} breathing	903	B_{2u}	6	BO_3^{3-} breathing
905	B_{3u}	397	BO_3^{3-} breathing	903	B_{3u}	0	BO_3^{3-} breathing
904	B_{1u}	5	BO_3^{3-} breathing	752	B_{3u}	1309	BO_3^{3-} out of plane
901	B_{2u}	6	BO_3^{3-} breathing	737	B_{1u}	11	BO_3^{3-} out of plane
880	B_{3u}	3914	'Hydride in plane stretching'*	736	B_{2u}	980	BO_3^{3-} out of plane
758	B_{3u}	1303	BO_3^{3-} out of plane	735	B_{3u}	0	BO_3^{3-} out of plane
741	B_{2u}	991	BO_3^{3-} out of plane	705	B_{1u}	2258	'Hydride in plane stretching'*
739	B_{1u}	167	BO_3^{3-} out of plane	689	B_{2u}	43	'Hydride in plane stretching'*
736	B_{3u}	235	BO_3^{3-} out of plane	627	B_{3u}	2041	'Hydride in plane stretching'*
735	B_{1u}	35	'Hydride out of plane stretching'*	595	B_{1u}	31	B-O bending
593	B_{2u}	1005	'Hydride out of plane stretching'*	591	B_{1u}	117	B-O bending
593	B_{1u}	39	B-O bending	586	B_{2u}	14	B-O bending
586	B_{1u}	13	B-O bending	583	B_{1u}	46	B-O bending

581	B_{1u}	46	B-O bending	580	B_{3u}	38	B-O bending
581	B_{3u}	9	B-O bending	579	B_{1u}	2	B-O bending
579	B_{2u}	496	B-O bending	575	B_{2u}	7	B-O bending
575	B_{2u}	50	B-O bending	574	B_{2u}	120	B-O bending
572	B_{1u}	184	B-O bending	569	B_{3u}	30	B-O bending
569	B_{3u}	30	B-O bending	564	B_{2u}	50	B-O bending
567	B_{2u}	137	B-O bending	511	B_{1u}	109	‘Hydride out of plane stretching’*
546	B_{2u}	1627	‘Hydride out of plane stretching’*	403	B_{2u}	1286	‘Hydride out of plane stretching’*
320	B_{3u}	117	BO_3^{3-} rocking, coupled with Sr atoms	319	B_{3u}	123	BO_3^{3-} rocking, coupled with Sr atoms
310	B_{1u}	211	BO_3^{3-} rocking, coupled with Sr atoms	309	B_{1u}	207	BO_3^{3-} rocking, coupled with Sr atoms
309	B_{2u}	4	BO_3^{3-} rocking, coupled with Sr atoms	308	B_{2u}	2	BO_3^{3-} rocking, coupled with Sr atoms
301	B_{2u}	5	BO_3^{3-} rocking, coupled with Sr atoms	298	B_{2u}	11	BO_3^{3-} rocking, coupled with Sr atoms
291	B_{3u}	4	BO_3^{3-} rocking, coupled with Sr atoms	291	B_{3u}	4	BO_3^{3-} rocking, coupled with Sr atoms
286	B_{2u}	187	BO_3^{3-} rocking, coupled with Sr atoms	286	B_{2u}	202	BO_3^{3-} rocking, coupled with Sr atoms
285	B_{1u}	632	BO_3^{3-} rocking, coupled with Sr atoms	285	B_{1u}	632	BO_3^{3-} rocking, coupled with Sr atoms
270	B_{1u}	774	BO_3^{3-} rocking, coupled with Sr atoms	270	B_{1u}	759	BO_3^{3-} rocking, coupled with Sr atoms
270	B_{3u}	5	BO_3^{3-} rocking, coupled with Sr atoms	269	B_{3u}	4.	BO_3^{3-} rocking, coupled with Sr atoms
256	B_{1u}	956	BO_3^{3-} rocking, coupled with Sr atoms	256	B_{1u}	961	BO_3^{3-} rocking, coupled with Sr atoms
249	B_{2u}	1733	BO_3^{3-} rocking, coupled with Sr atoms	249	B_{2u}	1728	BO_3^{3-} rocking, coupled with Sr atoms
245	B_{3u}	117	BO_3^{3-} rocking, coupled with Sr atoms	245	B_{3u}	117	BO_3^{3-} rocking, coupled with Sr atoms
242	B_{2u}	291	BO_3^{3-} rocking, coupled with Sr atoms	242	B_{2u}	292	BO_3^{3-} rocking, coupled with Sr atoms
228	B_{2u}	769	BO_3^{3-} rocking, coupled with Sr atoms	228	B_{1u}	440	BO_3^{3-} rocking, coupled with Sr atoms
228	B_{1u}	432	BO_3^{3-} rocking, coupled with Sr atoms	227	B_{2u}	810	BO_3^{3-} rocking, coupled with Sr atoms
225	B_{3u}	52	BO_3^{3-} rocking, coupled with Sr atoms	225	B_{3u}	48	BO_3^{3-} rocking, coupled with Sr atoms
218	B_{3u}	509	BO_3^{3-} rocking, coupled with Sr atoms	217	B_{3u}	516	BO_3^{3-} rocking, coupled with Sr atoms
217	B_{2u}	427	BO_3^{3-} rocking, coupled with Sr atoms	216	B_{2u}	494	BO_3^{3-} rocking, coupled with Sr atoms
210	B_{1u}	252	BO_3^{3-} rocking, coupled with Sr atoms	209	B_{1u}	251	BO_3^{3-} rocking, coupled with Sr atoms
205	B_{1u}	112	BO_3^{3-} rocking, coupled with Sr atoms	204	B_{1u}	110	BO_3^{3-} rocking, coupled with Sr atoms
202	B_{3u}	955	BO_3^{3-} rocking, coupled with Sr atoms	202	B_{3u}	954	BO_3^{3-} rocking, coupled with Sr atoms
201	B_{2u}	207	BO_3^{3-} rocking, coupled with Sr atoms	200	B_{2u}	152	BO_3^{3-} rocking, coupled with Sr atoms
192	B_{2u}	323	BO_3^{3-} rocking, coupled with Sr atoms	192	B_{2u}	322	BO_3^{3-} rocking, coupled with Sr atoms
183	B_{1u}	48	BO_3^{3-} rocking, coupled with Sr atoms	183	B_{1u}	52	BO_3^{3-} rocking, coupled with Sr atoms
183	B_{3u}	74	BO_3^{3-} rocking, coupled with Sr atoms	183	B_{3u}	76	BO_3^{3-} rocking, coupled with Sr atoms
180	B_{1u}	250	BO_3^{3-} rocking, coupled with Sr atoms	180	B_{1u}	238	BO_3^{3-} rocking, coupled with Sr atoms
179	B_{3u}	1771	BO_3^{3-} rocking, coupled with Sr atoms	179	B_{3u}	1762	BO_3^{3-} rocking, coupled with Sr atoms
168	B_{1u}	585	BO_3^{3-} rocking, coupled with Sr atoms	168	B_{1u}	585	BO_3^{3-} rocking, coupled with Sr atoms
157	B_{1u}	50	BO_3^{3-} rocking, coupled with Sr atoms	157	B_{1u}	49	BO_3^{3-} rocking, coupled with Sr atoms
157	B_{2u}	50	BO_3^{3-} rocking, coupled with Sr atoms	157	B_{2u}	51	lattice low frequency vibrations
156	B_{3u}	634	lattice low frequency vibrations	156	B_{3u}	636	lattice low frequency vibrations
149	B_{1u}	107	lattice low frequency vibrations	148	B_{1u}	116	lattice low frequency vibrations
146	B_{2u}	137	lattice low frequency vibrations	146	B_{2u}	141	lattice low frequency vibrations
138	B_{2u}	9	lattice low frequency vibrations	138	B_{2u}	10	lattice low frequency vibrations
130	B_{1u}	73	lattice low frequency vibrations	130	B_{1u}	71	lattice low frequency vibrations
123	B_{2u}	34	lattice low frequency vibrations	123	B_{2u}	37	lattice low frequency vibrations
122	B_{3u}	62	lattice low frequency vibrations	122	B_{3u}	62	lattice low frequency vibrations
113	B_{1u}	6	lattice low frequency vibrations	113	B_{1u}	6	lattice low frequency vibrations
112	B_{2u}	4	lattice low frequency vibrations	111	B_{2u}	3	lattice low frequency vibrations
110	B_{3u}	3	lattice low frequency vibrations	110	B_{3u}	4	lattice low frequency vibrations
106	B_{2u}	82	lattice low frequency vibrations	106	B_{2u}	82	lattice low frequency vibrations
102	B_{3u}	29	lattice low frequency vibrations	102	B_{3u}	29	lattice low frequency vibrations
101	B_{2u}	1	lattice low frequency vibrations	101	B_{2u}	1	lattice low frequency vibrations
95	B_{1u}	4	lattice low frequency vibrations	95	B_{1u}	5	lattice low frequency vibrations
88	B_{1u}	66	lattice low frequency vibrations	87	B_{1u}	66	lattice low frequency vibrations
84	B_{3u}	139	lattice low frequency vibrations	84	B_{3u}	139	lattice low frequency vibrations
61	B_{3u}	92	lattice low frequency vibrations	61	B_{3u}	92	lattice low frequency vibrations

Table S6: Vibrational Raman data calculated by quantum chemical means. The frequency, irreducible representation, intensity and assignment to vibrations are given within this table for $Sr_5(^{nat}BO_3)_3H$ and $Sr_5(^{11}BO_3)_3D$. Vibrations marked with and asterisk are graphically visualized in figure S18.

$Sr_5(BO_3)_3H$				$Sr_5(^{11}BO_3)_3D$			
**Frequ-ency [cm^{-1}]**	Γ_{irrep}	**Intensity [km/mol]**	**Assignment**	**Frequ-ency [cm^{-1}]**	Γ_{irrep}	**Intensity [km/mol]**	**Assignment**
1276	B_{3g}	0	B-O stretching	1266	B_{3g}	0	B-O stretching
1270	A_g	8	B-O stretching	1261	A_g	4	B-O stretching
1269	B_{3g}	1	B-O stretching	1256	B_{3g}	1	B-O stretching
1257	A_g	36	B-O stretching	1249	A_g	18	B-O stretching
1238	B_{3g}	1	B-O stretching	1228	B_{3g}	1	B-O stretching
1236	B_{1g}	6	B-O stretching	1226	B_{2g}	0	B-O stretching
1235	B_{2g}	1	B-O stretching	1226	B_{1g}	3	B-O stretching
1224	A_g	5	B-O stretching	1215	A_g	2	B-O stretching
1211	B_{2g}	0	B-O stretching	1203	B_{2g}	0	B-O stretching
1208	B_{3g}	33	B-O stretching	1199	B_{3g}	13	B-O stretching
1205	B_{1g}	4	B-O stretching	1196	B_{1g}	2	B-O stretching
1202	A_g	8	B-O stretching	1194	A_g	4	B-O stretching
999	B_{3g}	34	'Hydride in plane stretching'*	922	A_g	137	BO_3^{3-} breathing
972	A_g	94	'Hydride in plane stretching'*	920	B_{3g}	0	BO_3^{3-} breathing
927	B_{1g}	3	'Hydride in plane stretching'*	907	B_{3g}	0	BO_3^{3-} breathing
921	A_g	211	BO_3^{3-} breathing	907	B_{2g}	2	BO_3^{3-} breathing
920	B_{3g}	0	BO_3^{3-} breathing	906	A_g	317	BO_3^{3-} breathing
909	B_{2g}	0	BO_3^{3-} breathing	906	B_{1g}	5	BO_3^{3-} breathing
907	B_{3g}	0	BO_3^{3-} breathing	767	B_{1g}	1	BO_3^{3-} out of plane
906	A_g	400	BO_3^{3-} breathing	760	B_{2g}	5	BO_3^{3-} out of plane
906	B_{1g}	12	BO_3^{3-} breathing	759	B_{3g}	0	BO_3^{3-} out of plane
862	B_{2g}	99	'Hydride in plane stretching'*	756	A_g	2	BO_3^{3-} out of plane
764	B_{3g}	0	BO_3^{3-} out of plane	755	B_{1g}	0	BO_3^{3-} out of plane
763	B_{1g}	4	BO_3^{3-} out of plane	751	B_{2g}	0	BO_3^{3-} out of plane
760	A_g	2	BO_3^{3-} out of plane	715	B_{3g}	12	'Hydride in plane stretching'*
760	B_{2g}	0	BO_3^{3-} out of plane	690	A_g	31	'Hydride in plane stretching'*
759	B_{1g}	2	BO_3^{3-} out of plane	658	B_{1g}	2	'Hydride in plane stretching'*
756	B_{2g}	1	BO_3^{3-} out of plane	619	B_{2g}	40	'Hydride in plane stretching'*
725	B_{3g}	26	BO_3^{3-} out of plane	595	B_{3g}	2	B-O bending
595	B_{3g}	20	B-O bending	592	B_{3g}	11	B-O bending
593	A_g	17	B-O bending	591	A_g	2	B-O bending
588	B_{1g}	12	B-O bending	587	B_{1g}	6	B-O bending
587	B_{3g}	8	B-O bending	582	A_g	49	B-O bending
585	B_{2g}	19	B-O bending	581	B_{3g}	6	B-O bending
583	A_g	156	B-O bending	576	B_{2g}	0	B-O bending
581	B_{3g}	9	B-O bending	572	A_g	15	B-O bending
575	A_g	464	B-O bending	571	B_{3g}	3	B-O bending
573	B_{1g}	9	B-O bending	569	B_{1g}	4	B-O bending
571	B_{3g}	12	B-O bending	564	A_g	4	B-O bending
569	A_g	131	B-O bending	564	B_{2g}	4	B-O bending
567	B_{2g}	16	B-O bending	514	B_{3g}	9	'Hydride out of plane stretching'*
552	A_g	1000	'Hydride out of plane stretching'*	402	A_g	797	'Hydride out of plane stretching'*
319	B_{1g}	26	BO_3^{3-} rocking, coupled with Sr atoms	318	B_{1g}	12	BO_3^{3-} rocking, coupled with Sr atoms
304	B_{2g}	24	BO_3^{3-} rocking, coupled with Sr atoms	303	B_{2g}	11	BO_3^{3-} rocking, coupled with Sr atoms
295	B_{3g}	33	BO_3^{3-} rocking, coupled with Sr atoms	294	B_{3g}	13	BO_3^{3-} rocking, coupled with Sr atoms
290	A_g	274	BO_3^{3-} rocking, coupled with Sr atoms	290	A_g	119	BO_3^{3-} rocking, coupled with Sr atoms
283	B_{2g}	100	BO_3^{3-} rocking, coupled with Sr atoms	283	B_{2g}	45	BO_3^{3-} rocking, coupled with Sr atoms
280	B_{3g}	93	BO_3^{3-} rocking, coupled with Sr atoms	279	B_{3g}	41	BO_3^{3-} rocking, coupled with Sr atoms
277	B_{1g}	35	BO_3^{3-} rocking, coupled with Sr atoms	277	B_{1g}	16	BO_3^{3-} rocking, coupled with Sr atoms
274	B_{2g}	0	BO_3^{3-} rocking, coupled with Sr atoms	274	B_{2g}	0	BO_3^{3-} rocking, coupled with Sr atoms
270	B_{3g}	63	BO_3^{3-} rocking, coupled with Sr atoms	270	B_{3g}	31	BO_3^{3-} rocking, coupled with Sr atoms
268	A_g	41	BO_3^{3-} rocking, coupled with Sr atoms	268	A_g	33	BO_3^{3-} rocking, coupled with Sr atoms
267	B_{1g}	0	BO_3^{3-} rocking, coupled with Sr atoms	267	B_{1g}	0	BO_3^{3-} rocking, coupled with Sr atoms
256	B_{3g}	3	BO_3^{3-} rocking, coupled with Sr atoms	255	B_{3g}	1	BO_3^{3-} rocking, coupled with Sr atoms
252	A_g	36	BO_3^{3-} rocking, coupled with Sr atoms	252	A_g	29	BO_3^{3-} rocking, coupled with Sr atoms
251	B_{2g}	11	BO_3^{3-} rocking, coupled with Sr atoms	251	B_{2g}	5	BO_3^{3-} rocking, coupled with Sr atoms
245	A_g	15	BO_3^{3-} rocking, coupled with Sr atoms	244	A_g	13	BO_3^{3-} rocking, coupled with Sr atoms
243	B_{1g}	19	BO_3^{3-} rocking, coupled with Sr atoms	243	B_{1g}	9.	BO_3^{3-} rocking, coupled with Sr atoms
237	B_{1g}	23	BO_3^{3-} rocking, coupled with Sr atoms	237	B_{1g}	10	BO_3^{3-} rocking, coupled with Sr atoms
232	A_g	41	BO_3^{3-} rocking, coupled with Sr atoms	232	A_g	37	BO_3^{3-} rocking, coupled with Sr atoms
225	B_{2g}	22	BO_3^{3-} rocking, coupled with Sr atoms	225	B_{2g}	10	BO_3^{3-} rocking, coupled with Sr atoms
224	B_{3g}	130	BO_3^{3-} rocking, coupled with Sr atoms	223	B_{3g}	62	BO_3^{3-} rocking, coupled with Sr atoms
220	A_g	21	BO_3^{3-} rocking, coupled with Sr atoms	220	B_{1g}	27	BO_3^{3-} rocking, coupled with Sr atoms

220	B_{1g}	60	BO_3^{3-} rocking, coupled with Sr atoms	220	A_g	10	BO_3^{3-} rocking, coupled with Sr atoms
213	B_{2g}	0	BO_3^{3-} rocking, coupled with Sr atoms	212	B_{2g}	0	BO_3^{3-} rocking, coupled with Sr atoms
212	B_{3g}	161	BO_3^{3-} rocking, coupled with Sr atoms	211	B_{3g}	70	BO_3^{3-} rocking, coupled with Sr atoms
207	B_{1g}	8	BO_3^{3-} rocking, coupled with Sr atoms	207	B_{1g}	3	BO_3^{3-} rocking, coupled with Sr atoms
207	A_g	1	BO_3^{3-} rocking, coupled with Sr atoms	206	A_g	1	BO_3^{3-} rocking, coupled with Sr atoms
206	B_{2g}	16	BO_3^{3-} rocking, coupled with Sr atoms	205	B_{2g}	7	BO_3^{3-} rocking, coupled with Sr atoms
200	A_g	67	BO_3^{3-} rocking, coupled with Sr atoms	200	A_g	57	BO_3^{3-} rocking, coupled with Sr atoms
197	B_{3g}	98	BO_3^{3-} rocking, coupled with Sr atoms	197	B_{3g}	45	BO_3^{3-} rocking, coupled with Sr atoms
195	B_{3g}	13	BO_3^{3-} rocking, coupled with Sr atoms	194	B_{3g}	5	BO_3^{3-} rocking, coupled with Sr atoms
191	B_{1g}	2	BO_3^{3-} rocking, coupled with Sr atoms	191	B_{1g}	1	BO_3^{3-} rocking, coupled with Sr atoms
185	B_{2g}	18	BO_3^{3-} rocking, coupled with Sr atoms	185	B_{2g}	8	BO_3^{3-} rocking, coupled with Sr atoms
176	B_{3g}	3	BO_3^{3-} rocking, coupled with Sr atoms	176	B_{3g}	1	BO_3^{3-} rocking, coupled with Sr atoms
175	B_{2g}	0	BO_3^{3-} rocking, coupled with Sr atoms	175	B_{2g}	0	BO_3^{3-} rocking, coupled with Sr atoms
175	A_g	21	BO_3^{3-} rocking, coupled with Sr atoms	175	A_g	8	BO_3^{3-} rocking, coupled with Sr atoms
173	B_{3g}	1	BO_3^{3-} rocking, coupled with Sr atoms	172	B_{3g}	0	BO_3^{3-} rocking, coupled with Sr atoms
168	B_{2g}	11	BO_3^{3-} rocking, coupled with Sr atoms	168	B_{2g}	5	BO_3^{3-} rocking, coupled with Sr atoms
161	A_g	50	BO_3^{3-} rocking, coupled with Sr atoms	161	A_g	31	BO_3^{3-} rocking, coupled with Sr atoms
155	B_{3g}	166	BO_3^{3-} rocking, coupled with Sr atoms	155	B_{2g}	17	lattice low frequency vibrations
155	B_{2g}	38	BO_3^{3-} rocking, coupled with Sr atoms	154	B_{3g}	73	lattice low frequency vibrations
151	A_g	57	lattice low frequency vibrations	151	A_g	47	lattice low frequency vibrations
148	B_{3g}	2	lattice low frequency vibrations	147	B_{3g}	2	lattice low frequency vibrations
143	B_{1g}	6	lattice low frequency vibrations	142	B_{1g}	3	lattice low frequency vibrations
139	B_{3g}	4	lattice low frequency vibrations	139	B_{3g}	2	lattice low frequency vibrations
133	A_g	18	lattice low frequency vibrations	132	A_g	15	lattice low frequency vibrations
130	B_{2g}	18	lattice low frequency vibrations	130	B_{2g}	8	lattice low frequency vibrations
129	B_{1g}	2	lattice low frequency vibrations	129	B_{1g}	1	lattice low frequency vibrations
128	B_{1g}	34	lattice low frequency vibrations	128	B_{1g}	15	lattice low frequency vibrations
125	B_{3g}	3	lattice low frequency vibrations	125	B_{3g}	2	lattice low frequency vibrations
124	B_{1g}	18	lattice low frequency vibrations	124	B_{1g}	8	lattice low frequency vibrations
119	A_g	88	lattice low frequency vibrations	119	A_g	46	lattice low frequency vibrations
110	A_g	14	lattice low frequency vibrations	110	A_g	6	lattice low frequency vibrations
108	B_{3g}	0	lattice low frequency vibrations	108	B_{3g}	0	lattice low frequency vibrations
102	B_{1g}	3	lattice low frequency vibrations	102	B_{1g}	1	lattice low frequency vibrations
101	B_{2g}	5	lattice low frequency vibrations	101	B_{2g}	2	lattice low frequency vibrations
100	A_g	112	lattice low frequency vibrations	100	A_g	61	lattice low frequency vibrations
99	B_{2g}	10	lattice low frequency vibrations	99	B_{2g}	5	lattice low frequency vibrations
96	B_{1g}	15	lattice low frequency vibrations	96	B_{1g}	7	lattice low frequency vibrations
88	B_{2g}	12	lattice low frequency vibrations	88	B_{2g}	6	lattice low frequency vibrations
84	B_{2g}	119	lattice low frequency vibrations	84	B_{2g}	54	lattice low frequency vibrations
79	B_{1g}	109	lattice low frequency vibrations	79	B_{1g}	49	lattice low frequency vibrations
75	A_g	30	lattice low frequency vibrations	75	A_g	21	lattice low frequency vibrations
75	B_{3g}	6	lattice low frequency vibrations	75	B_{3g}	3	lattice low frequency vibrations
57	A_g	71	lattice low frequency vibrations	57	A_g	54	lattice low frequency vibrations
55	B_{1g}	84	lattice low frequency vibrations	55	B_{1g}	38	lattice low frequency vibrations
54	B_{3g}	8	lattice low frequency vibrations	54	B_{3g}	4	lattice low frequency vibrations

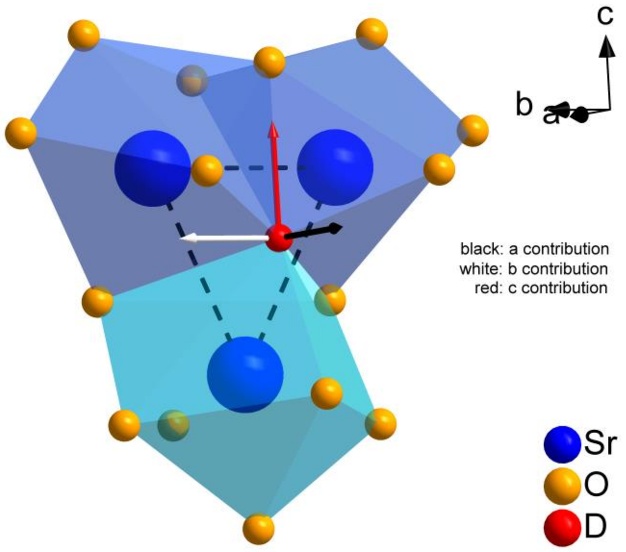

Figure S18: Hydride and deuteride vibrations deduced from quantum chemical calculations in Table S5 and S6. Two main vibrations occur in IR and Raman spectra: First, the black arrow depicts the movement that is perpendicular to the plane of the three strontium atoms forming a triangle, hereon called 'hydride out-of-plane mode' (2x Sr2, dark blue, and 1x Sr1, light blue). The other movement is parallel to the plane of the Sr_3 triangle (white and red arrow), herein called 'hydride in-plane mode'.

Borate groups assignment to the spectra in figures 5 and 6 of the main text

Figure 5:

The BO_3^{3-} stretching modes are located at 1350 – 1050 cm^{-1} and strongly visible in the experimental spectra. At around 900 cm^{-1}, calculations suggest the presence of breathing modes of BO_3^{3-}, which have smaller intensities in the calculated spectra compared to the experiment (the calculated IR transmittances are for an ideal single crystal and may therefore show some differences to the experimental data). Borate out-of-plane vibrational and B-O bending modes can be found at 750 cm^{-1} and 580 cm^{-1}, respectively.

Figure 6:

From 50 cm^{-1} to about 320 cm^{-1} low energy lattice vibrations and borate rocking modes are observed (blue labels). At around 600 cm^{-1}, a B-O bending mode is visible. Little intensity in the experimental is observed for the borate out-of-plane bending, in agreement with theoretical expectations due to symmetry constraints. (Note, that these modes are described for the plane of the borate groups). At 910 cm^{-1}, breathing modes of the BO_3^{3-} groups show resonances in good agreement to the experiment. Similar to the IR spectra, we do not discuss the details for the ^{nat}B - ^{11}B exchange, since the changes in the vibrational energies are minor compared to the hydride-deuteride shift.

Elemental analysis

Elemental analysis has been conducted on a Vario El microanalyzer with the $Sr_5(^{11}BO_3)_3D$ sample and its deuterium content amounts to 0.26 wt%. Full occupation of the 4c Wyckoff site is assumed for D. The experimentally determined deuterium content is close to the theoretical value of 0.32 wt%.

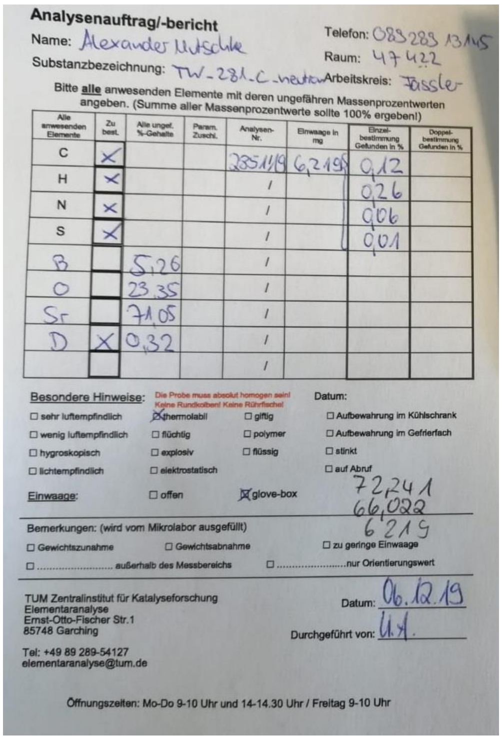
Analysenauftrag/-bericht

Name: Alexander Mutschke   Telefon: 089 289 13145

Substanzbezeichnung: TW-281-C-neutron   Raum: 47 422

Arbeitskreis: Fässler

Bitte alle anwesenden Elemente mit deren ungefähren Massenprozentwerten angeben. (Summe aller Massenprozentwerte sollte 100% ergeben!)

Alle anwesenden Elemente	Zu best.	Alle ungef. %-Gehalte	Param. Zuschl.	Analysen-Nr.	Einwaage in mg	Einzel-bestimmung Gefunden in %	Doppel-bestimmung Gefunden in %
C	X			2351/19	6,219	0,12	
H	X			/		0,26	
N	X			/		0,06	
S	X			/		0,01	
B		5,26		/			
O		23,35		/			
Sr		71,05		/			
D	X	0,32		/			
				/			

Besondere Hinweise: Die Probe muss absolut homogen sein! Keine Rundkolben! Keine Rührfische!   Datum:

☐ sehr luftempfindlich ☒ thermolabil ☐ giftig ☐ Aufbewahrung im Kühlschrank

☐ wenig luftempfindlich ☐ flüchtig ☐ polymer ☐ Aufbewahrung im Gefrierfach

☐ hygroskopisch ☐ explosiv ☐ flüssig ☐ stinkt

☐ lichtempfindlich ☐ elektrostatisch ☐ auf Abruf

Einwaage: ☐ offen ☒ glove-box   72,241 66,022 6,219

Bemerkungen: (wird vom Mikrolabor ausgefüllt)

☐ Gewichtszunahme ☐ Gewichtsabnahme ☐ zu geringe Einwaage

☐ .... außerhalb des Messbereichs ☐ ....nur Orientierungswert

TUM Zentralinstitut für Katalyseforschung
Elementaranalyse
Ernst-Otto-Fischer Str.1
85748 Garching

Tel: +49 89 289-54127
elementaranalyse@tum.de

Datum: 06.12.19

Durchgeführt von: U.A.

Öffnungszeiten: Mo-Do 9-10 Uhr und 14-14.30 Uhr / Freitag 9-10 Uhr

Figure S19: Elemental analysis of $Sr_5(^{11}BO_3)_3D$.

Full computational details

The solid-state NMR shielding tensors of $Sr_5(BO_3)_3H$ and hypothetical $Sr_5(BO_3)_3OH$ were calculated with the DFT-PBE method[2] using the CASTEP program package and the GIPAW formalism as implemented in CASTEP-NMR[3]. Ultrasoft pseudopotentials generated with the on-the-fly scheme[4] and a plane-wave basis set cut-off of 630 eV were applied. The reciprocal space was sampled using a 3×2×3 Monkhorst-Pack-type *k*-mesh.[5] The NMR shielding tensor of $Sr_5(BO_3)_3H$ was calculated both at the experimental geometry and DFT-PBE optimized geometry. In the geometry optimization, both the lattice parameters and atomic positions were fully optimized with a total energy convergence criterion of 0.5×10^{-5} eV/atom. The optimized lattice parameters *a*, *b*, and *c* of $Sr_5(BO_3)_3H$ differed from the experimental parameters by +1.1%, –0.4%, and +0.2%, respectively. The NMR shielding tensor of the hypothetical $Sr_5(BO_3)_3OH$ was calculated at the DFT-PBE0/TZVP-optimized geometry (see below). Molecular $SiMe_4$ was used as a reference system for calculating the ^1H NMR shifts. The calculations on $SiMe_4$ were carried out in a primitive cubic cell (*a* = 15 Å) using a plane-wave basis set cut-off of 700 eV and Γ-point for reciprocal space sampling. The structure of the $SiMe_4$ molecule was relaxed within the T_d point group. The isotropic ^1H shielding of $SiMe_4$ is 31.01 ppm. The isotropic ^1H shielding of the hydride in $Sr_5(BO_3)_3H$ is 25.09 ppm at the experimental geometry and 25.10 ppm at the optimized geometry. Both values lead in identical ^1H chemical shift of 5.9 ppm. The isotropic ^1H shielding in the hypothetical $Sr_5(BO_3)_3OH$ ($P2_12_12_1$) is 27.08 ppm, leading in a ^1H chemical shift of 3.9 ppm.

We also investigated $Sr_5(BO_3)_3H$, $Sr_5(^{11}BO_3)_3D$, and hypothetical $Sr_5(^{11}BO_3)_3OH$ using the CRYSTAL17 program package.[6] PBE0 hybrid density functional method and Gaussian-type basis sets were used.[7] The basis sets for Sr, O, and H have been previously derived from the molecular Karlsruhe def2 basis sets.[8] The basis set used for B is described in detail below. Polarized triple-zeta-valence (TZVP) basis sets were used for H, O, and B, polarized split-valence basis set for Sr.[9] The reciprocal space was sampled using a 4×2×3 Monkhorst-Pack-type *k*-mesh.[5] For the evaluation of the Coulomb and exchange integrals (TOLINTEG), tight tolerance factors of 8, 8, 8, 8, and 16 were used. Both the atomic positions and lattice constants were fully optimized within the constraints imposed by the space group symmetry. In the case of $Sr_5(BO_3)_3H$, the optimized lattice parameters *a*, *b*, and *c* differed from the experimental parameters by 0.0%, –0.3%, and –0.2%, respectively. The harmonic vibrational frequencies and IR intensities were obtained by using the computational schemes implemented in CRYSTAL.[10] $Sr_5(BO_3)_3H$ and $Sr_5(BO_3)_3D$ were confirmed to be true local minima with no imaginary frequencies. Hypothetical $Sr_5(BO_3)_3OH$ showed imaginary frequencies when optimized in the space group *Pnma*. Distorting the geometry along the first imaginary mode (92*i* cm^{-1}) decreased the symmetry to $P2_12_12_1$. In this space group, the optimized structure is a true local minimum (full structural details report below).

The wavenumbers of the IR and Raman spectra have been scaled by a factor of 0.96 to account for the overestimation typical for predicted harmonic frequencies (see e.g. A. P. Scott, L. Radom *J. Phys. Chem.* **1996**, *100*, 16502-16513 and http://cccbdb.nist.gov/). The final IR spectra were obtained by using Lorentzian peak profile with FWHM of 8 cm^{-1}. The Raman intensities have been calculated for a polycrystalline powder sample (total isotropic intensity in arbitrary units). When simulating the Raman spectrum, the temperature and laser wavelength were set to values corresponding to the experimental setup (T = 298.15 K, λ = 532 nm). The final spectrum was obtained by using pseudo-Voigt peak profile (50:50 Lorentzian: Gaussian) and FWHM of 8 cm^{-1}.

Basis set details for B

We modified the molecular def2-TZVP basis by removing the outermost s-function with an exponent of 0.06, leaving still three s-functions in the valence space. The exponents of the outermost s and p functions were set fixed to 0.17 and the exponents of the other s and p functions in the valence space were reoptimized for the boron atom in its ground state. Finally, the outermost s and p functions were combined into a single sp-type function to increase the efficiency of the CRYSTAL code. The resulting energy loss w.r.t the unmodified basis set is 15 mH. The full basis set is listed below in CRYSTAL input format:

```
5 7
0 0 6 2.0 1.0
  8564.8660687       0.22837198155E-03
  1284.1516263       0.17682576447E-02
  292.27871604       0.91407080516E-02
  82.775469176       0.36342638989E-01
  27.017939269       0.11063458441
  9.8149619660       0.23367344321
0 0 2 2.0 1.0
  3.9295537119       0.41818777978
  1.6980409139       0.22325473798
0 0 1 0.0 1.0
 0.57483069645       1.0000000000
0 1 1 0.0 1.0
 0.17000000000       1.0 1.0
0 2 3 1.0 1.0
  28.577505088       0.50265575179E-02
  6.7104492541       0.32801738965E-01
```

```
  2.0721954358       0.13151230768
0 2 1 0.0 1.0
 0.64922416350      1.0000000000
0 3 1 0.0 1.0
 0.50000000000      1.0000000000
```

Electronic structure of $Sr_5(BO_3)_3F$ and $Sr_5(BO_3)_3H$

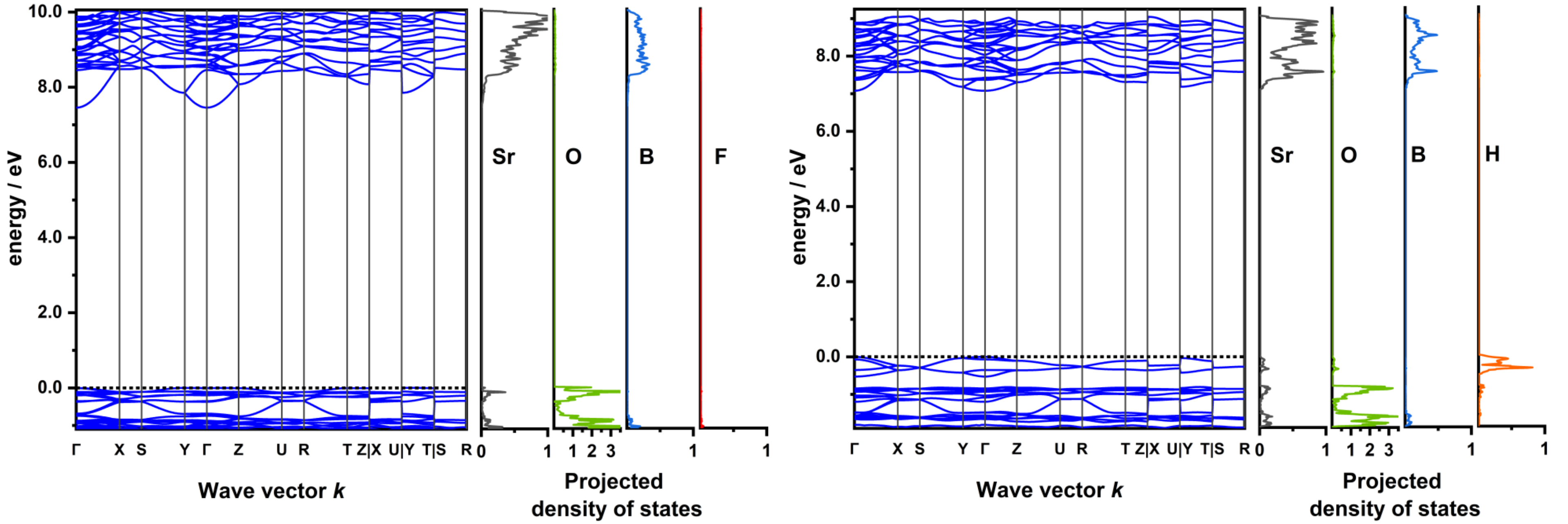

Figure S20: Band structure and projected density of states of $Sr_5(BO_3)_3F$ (left) and $Sr_5(BO_3)_3H$ (right) calculated at the DFT-PBE0/TZVP level of theory. An indirect bandgap of approx. 7.5 eV for the fluoride compound and ca. 7.0 eV for the hydride compound is obtained indicating insulating materials. The energy scale for $Sr_5(BO_3)_3H$ (right) has been shifted in such a way that the band level of the O 2p orbitals in both graphs are visually on the same level for better comparison. Interestingly, the hydride ion has a large impact on the highest-energy valence bands in the density of states analysis, resulting in a reduced bandgap in the hydride species. The band paths in the reciprocal space were obtained from the SeeK-path web service.[11]

Optimized structures in CIF format (DFT-PBE0/TZVP level of theory)

```
data_Sr5_BO3_3_H
_audit_creation_method FINDSYM

_cell_length_a    7.2013607500
_cell_length_b    14.1085813100
_cell_length_c    9.8041155600
_cell_angle_alpha 90.0000000000
_cell_angle_beta  90.0000000000
_cell_angle_gamma 90.0000000000

_symmetry_space_group_name_H-M "P 21/n 21/m 21/a"
_symmetry_Int_Tables_number 62
_space_group.reference_setting '062:-P 2ac 2n'
_space_group.transform_Pp_abc a,b,c;0,0,0

loop_
_space_group_symop_id
_space_group_symop_operation_xyz
1 x,y,z
2 x+1/2,-y+1/2,-z+1/2
3 -x,y+1/2,-z
4 -x+1/2,-y,z+1/2
5 -x,-y,-z
6 -x+1/2,y+1/2,z+1/2
7 x,-y+1/2,z
8 x+1/2,y,-z+1/2

loop_
_atom_site_label
_atom_site_type_symbol
_atom_site_symmetry_multiplicity
_atom_site_Wyckoff_label
_atom_site_fract_x
_atom_site_fract_y
_atom_site_fract_z
_atom_site_occupancy
Sr1 Sr   8 d 0.02887 0.11255 0.25356 1.00000
O1  O    8 d 0.09710 0.59102 0.15210 1.00000
B1  B    8 d 0.20101 0.53879 0.06109 1.00000
Sr2 Sr   8 d 0.25406 0.62039 0.37444 1.00000
O2  O    8 d 0.27778 0.06849 0.42767 1.00000
O3  O    8 d 0.28041 0.04495 0.10319 1.00000
O4  O    4 c 0.11557 0.25000 0.04406 1.00000
H1  H    4 c 0.12436 0.25000 0.73570 1.00000
O5  O    4 c 0.28425 0.25000 0.25172 1.00000
B2  B    4 c 0.28279 0.25000 0.11069 1.00000
Sr3 Sr   4 c 0.28893 0.25000 0.52137 1.00000
O6  O    4 c 0.44483 0.25000 0.03810 1.00000



data_Sr5_BO3_3_OH
_audit_creation_method FINDSYM

_cell_length_a    7.2244772000
_cell_length_b    14.0317010200
_cell_length_c    9.8413398100
_cell_angle_alpha 90.0000000000
_cell_angle_beta  90.0000000000
_cell_angle_gamma 90.0000000000

_symmetry_space_group_name_H-M "P 21 21 21"
_symmetry_Int_Tables_number 19
_space_group.reference_setting '019:P 2ac 2ab'
_space_group.transform_Pp_abc a,b,c;0,0,0

loop_
_space_group_symop_id
_space_group_symop_operation_xyz
1 x,y,z
2 x+1/2,-y+1/2,-z
3 -x,y+1/2,-z+1/2
4 -x+1/2,-y,z+1/2

loop_
```

```
_atom_site_label
_atom_site_type_symbol
_atom_site_symmetry_multiplicity
_atom_site_Wyckoff_label
_atom_site_fract_x
_atom_site_fract_y
_atom_site_fract_z
_atom_site_occupancy
Sr1 Sr   4 a 0.02782  0.11114  -0.00637 1.00000
Sr2 Sr   4 a -0.02447 0.88661  0.49137  1.00000
O1  O    4 a 0.07655  0.60237  -0.08518 1.00000
O2  O    4 a 0.89465  0.41929  0.60930  1.00000
B1  B    4 a 0.18875  0.55043  0.82875  1.00000
B2  B    4 a 0.79000  0.46979  0.70160  1.00000
Sr3 Sr   4 a 0.26574  0.60792  0.13521  1.00000
Sr4 Sr   4 a 0.78313  0.36790  0.38247  1.00000
O3  O    4 a 0.26799  0.06258  0.16728  1.00000
O4  O    4 a 0.71773  -0.07722 0.30531  1.00000
O5  O    4 a 0.28726  0.05465  0.83880  1.00000
O6  O    4 a 0.72636  -0.03111 0.62335  1.00000
O7  O    4 a 0.11088  0.26138  0.79857  1.00000
O8  O    4 a 0.11531  0.26323  0.48381  1.00000
O9  O    4 a 0.28098  0.25061  0.00343  1.00000
B3  B    4 a 0.27781  0.25727  0.86330  1.00000
Sr5 Sr   4 a 0.28280  0.24259  0.26665  1.00000
O10 O    4 a 0.43897  0.26242  0.79081  1.00000
H1  H    4 a 0.48122  0.78093  0.00905  1.00000
```

References

[1] K. Momma and F. Izumi, "VESTA 3 for three-dimensional visualization of crystal, volumetric and morphology data," *J. Appl. Crystallogr.*, **2011**, 44, 1272-1276.

[2] J. Perdew, K. Burke, M. Ernzerhof, *Phys. Rev. Lett.,* **1996**, *77*, 3865-3868.

[3] a) S. J. Clark, M. D. Segall, C. J. Pickard, P. J. Hasnip, M. I. Probert, K. Refson, M. C. Payne, *Z. Kristallogr.* **2005**, *220*, 567-570; b) C. J. Pickard, F. Mauri, *Phys. Rev. B* **2001**, *63*, 245101; c) J. R. Yates, C. J. Pickard, F. Mauri, *Phys. Rev. B* **2007**, *76*, 024401.

[4] D. Vanderbilt, *Phys.Rev. B* **1990**, *41*, 7892-7895.

[5] H. J. Monkhorst, J. D. Pack, *Phys. Rev. B* **1976**, *13*, 5188-5192.

[6] R. Dovesi, A. Erba, R. Orlando, C. M. Zicovich-Wilson, B. Civalleri, L. Maschio, M. Rérat, S. Casassa, J. Baima, S. Salustro, B. Kirtman, *Wiley Interdiscip.Rev: Comput Mol. Sci.* **2018**, *8*, 1-36.

[7] C. Adamo, V. Barone, *Chem. Phys.* **1999**, *110*, 6158-6170.

[8] F. Weigend, R. Ahlrichs, *Phys. Chem. Chem. Phys.* **2005**, *7*, 3297-3305.

[9] a) A. J. Karttunen, T. Tynell, M. Karppinen, *J. Phys. Chem. C* **2015**, *119*, 13105-13114; b) B. Scheibe, C. Pietzonka, O. Mustonen, M. Karppinen, A. J. Karttunen, M. Atanasov, F. Neese, M. Conrad, F. Kraus, *Angew. Chem.* **2018**, *130*, 2964-2968.

[10] a) F. Pascale, C. M. Zicovich-Wilson, F. Lopez Gejo, B. Civalleri, R. Orlando, R. Dovesi, *Comput Chem.* **2004**, *25*, 888-897; b) C. Zicovich-Wilson, F. Pascale, C. Roetti, V. Saunders, R. Orlando, R. Dovesi, *Comput. Chem.* **2004**, *25*, 1873-1881; c) L. Maschio, B. Kirtman, R. Orlando, M. Rèrat, *J. Chem. Phys.* **2012**, *137*, 204113.

[11] a) Y. Hinuma, G. Pizzi, Y. Kumagai, F. Oba, I. Tanaka, *Comp. Mat. Sci.* **2017**, *128*, 140 b) A. Togo, I. Tanaka, "Spglib: a software library for crystal symmetry search", arXiv:1808.01590, **2018**
